# Restore axonal conductance in a locally demyelinated axon with electromagnetic stimulation

**DOI:** 10.1088/1741-2552/adb213

**Published:** 2025-02-14

**Authors:** Hui Ye, Yanan Chen, Ji Chen, Jenna Hendee

**Affiliations:** 1 Department of Biology, Loyola University Chicago, Chicago, IL, United States of America; 2 Department of Electrical and Computer Engineering, University of Houston, Houston, TX, United States of America

**Keywords:** myelinated axon, demyelination, axonal conductance, ion channel, microcoil stimulation, computational modeling

## Abstract

*Objective*. Axonal demyelination leads to failure of axonal conduction. Current research on demyelination focuses on the promotion of remyelination. Electromagnetic stimulation is widely used to promote neural activity. We hypothesized that electromagnetic stimulation of the demyelinated area, by providing excitation to the nodes of Ranvier, could rescue locally demyelinated axons from conductance failure. *Approach*. We built a multi-compartment NEURON model of a myelinated axon under electromagnetic stimulation. We simulated the action potential (AP) propagation and observed conductance failure when local demyelination occurred. Conductance failure was due to current leakage and a lack of activation of the nodes in the demyelinated region. To investigate the effects of electromagnetic stimulation on locally demyelinated axons, we positioned a miniature coil next to the affected area to activate nodes in the demyelinated region. *Main results*. Subthreshold microcoil stimulation caused depolarization of node membranes. This depolarization, in combination with membrane depolarization induced by the invading AP, resulted in sufficient activation of nodes in the demyelinated region and restoration of axonal conductance. Efficacy of restoration was dependent on the amplitude and frequency of the stimuli, and the location of the microcoil relative to the targeted nodes. The restored axonal conductance was due to the enhanced Na^+^ current and reduced K^+^ current in the nodes, rather than a reduction in leakage current in the demyelinated region. Finally, we found that microcoil stimulation had no effect on axonal conductance in healthy, myelinated axons. *Significance*. Activation of nodes in the demyelinated region using electromagnetic stimulation provides an alternative treatment strategy to restore axonal function under local demyelination conditions. Results provide insights to the development of microcoil technology for the treatment of focal segmental demyelination cases, such as neuropraxia, spinal cord injury, and auditory nerve demyelination.

**Abbreviations**
4-AP: 4-aminopyridine
AP: Action potential
CNS: Central nerve system
CMT: Charcot–Marie–Tooth disease
CIs: Cochlear implants
FH: Frankenhaeuser–Huxley
GBS: Guillain–Barré syndrome
*µ*MS: Micromagnetic stimulation
MS: Multiple sclerosis
NMOSD: Neuromyelitis optica spectrum disorder
PNS: Periphery nerve system
SCI: Spinal cord injury
SGN: Spiral ganglion neurons
TMS: Transcranial magnetic stimulation

## 1. Introduction

The fast and efficient transmission of electrical pulses in the nervous system is mediated by myelinated nerve fibers. Removal of the myelin sheath or demyelination leads to a delay, inconsistency, or failure of AP propagation in demyelinated axons. This accounts for the cellular mechanisms underlying CNS demyelinating diseases, such as MS and NMOSD, and PNS demyelinating diseases, such as GBS and CMT. Local neural injury can also lead to focal demyelination, as in the case of SCI when the local spine was crushed (Eftekharpour *et al* 2007, Liu *et al* 2013). Peripheral nerve injuries are commonly observed during sports and occupational activity. Shoulder dislocation can cause spinal accessory nerve injuries. Repetitive forearm pronation causes injury to the superficial branch of the radial nerve. Under mild injury (neuropraxia), these conditions are characterized by local damage to myelin fibers around the axon (Neal and Fields 2010).

The biophysics of axonal failure in demyelinated axons have been a central topic of demyelination studies for decades. Axonal failure in the demyelinated region is largely due to the failure of activation of the nodes of Ranvier by the invading AP. In a healthy, myelinated axon, the invading AP in the upstream node generates an axial current that depolarizes and activates the downstream node, which warrants the saltatory propagation of nerve pulses. When demyelination occurs, there is insufficient longitudinal current spreading in front of the impulse to the next node (Dale Purves *et al* 2011). Supporting this notion, about three-quarters of people with MS find their symptoms worsen in response to heat (the Uhthoff phenomenon (Frohman *et al* 2013)), as higher temperatures could shorten the invading AP and decrease the axial current. A shortened internode length, which allows less leakage current alongside increased axial current spreading and node activation, was observed in patients with MS when spontaneous remyelination occurred (Chari 2007).

Currently, there is no effective pharmacological treatment to completely restore the functionality of myelin and nerve conduction in demyelinated axons. Therefore, demyelination research that targets conductance failure focuses on ensuring a large axial current flow through the demyelinated region by *increasing node activity* or via *remyelination. Increasing node activity* can directly rescue axonal conductance during demyelination. Node excitability can be increased by pharmacological methods, such as 4-AP, a potassium channel blocker. *In vitro* studies have shown that 4-AP can improve the conduction of APs in demyelinated nerve fibers, thereby increasing the release of neurotransmitters in synapses and at neuromuscular junctions (Sherratt *et al* 1980, Bostock *et al* 1981). 4-AP is currently approved for the treatment of patients with MS and has been shown to improve walking ability (Goodman and Stone 2013, Baird *et al* 2018). Although pharmacological approaches that can enhance axonal conductance have significant benefits for the treatment of demyelinating diseases, these approaches are typically associated with adverse side effects. For example, 4-AP can cause paresthesia, dizziness, nausea/vomiting, etc (Jensen *et al* 2014).

*Remyelination* is the process of restoring demyelinated nerve fibers with new myelin (Franklin and Ffrench-Constant 2008). Remyelination can prevent the leakage of axial currents and restore axonal conductance. Several small molecules have been identified with the potential to promote remyelination by targeting diverse cellular and molecular pathways (Najm *et al* 2015, Rankin *et al* 2019, Chen *et al* 2021). These molecules typically enhance the differentiation of oligodendrocyte precursor cells into myelinating oligodendrocytes or protect remyelinating oligodendrocytes from an inflammatory environment. Stem cell transplantation could lead to remyelination, restoration of node structure, ion channel clustering, and functional recovery of axonal conductance in animal models of demyelination (Eftekharpour *et al* 2007, Ruff *et al* 2013). Electrical stimulation can also induce myelin formation by enhancing oligodendrocyte maturation (Lee *et al* 2017). Recently, electrical stimulation was used to restore the impaired myelin membrane in the mouse dorsal root ganglion via upregulation of lipid biosynthesis (Intisar *et al* 2022). Clinically, TMS can promote remyelination of neurons by activating axonal fibers and increasing the number of oligodendrocytes (Cullen *et al* 2019). In these examples, the electromagnetic fields were harnessed to promote remyelination.

To the best of our knowledge, there are no previous studies that have investigated whether electromagnetic stimulation of the demyelinated region can enhance node activation and rescue conductance failure after demyelination. However, several recent findings have suggested that such stimulation can be used to control and activate axonal elements, such as the nodes of Ranvier (Ye and Steiger 2015, Ye *et al* 2022b). For example, electromagnetic pulses cause immediate and transient activation of voltage-gated sodium channels in cultured neurons and acute rat brain slices (Banerjee *et al* 2017). Electromagnetic fields also affect potassium channels by changing their activation and inactivation properties (Tan *et al* 2013). Consequently, it is critical to investigate whether electromagnetic stimulation can be used to directly promote axonal conductance by activating nodes in the demyelinated region. In this proof-of-concept study, we tested the hypothesis that magnetic stimulation of the demyelinated area, by providing excitation to the nodes of Ranvier, could rescue locally demyelinated axons from conductance failure.

However, two technical challenges must be addressed. First, although it is possible to directly monitor node activity using advanced electrophysiology technology, such as patch clamp on the node (Kanda *et al* 2019), experimentally monitoring node activity is extremely challenging. This is because the externally applied electromagnetic field can interfere with the delicate node signal. Therefore, we performed computational simulations. We implement a multi-compartment model of a locally demyelinated axon under magnetic stimulation, using the NEURON simulation environment (Hines and Carnevale 1997).

Secondly, the externally applied electric field must be sufficiently focused on the demyelinated area. This could be achieved with either electrode or miniature coil. For electric stimulation, the electrodes were positioned close to the targeted neural tissue to deliver electric current. In magnetic stimulation, electric current is generated via electromagnetic induction, which does not require the stimulating coil to be in direct contact with the target tissue (Maccabee *et al* 1991, 1993, Ye *et al* 2010, 2011, Ye and Steiger 2015). This mitigates numerous problems that can arise at the brain–electrode interface, such as charge transfer, electrode surface modification, and corrosion (Polikov *et al* 2005, Cogan 2008, Koivuniemi *et al* 2011). Recent development of *µ*MS technology significantly improved the specificity of coil stimulation (Bonmassar *et al* 2012, Park *et al* 2013). These submillimeter coils are smaller than the dimension between two adjacent nodes on a single myelinated axon and can, therefore, be positioned to target a specific node. Furthermore, the coil can be implanted under the cover of soft biocompatible materials. This largely prevents the neural response to implantation (Saxena *et al* 2013, Canales *et al* 2015), including inflammatory and immune responses (Kim *et al* 2004, Lee *et al* 2016, Liu *et al* 2017). With such an implantation, focal stimulation of the targeted deep structure is feasible (Bonmassar *et al* 2012, Saha *et al* 2022b).

In this study, we applied electric stimulation using microcoil technology to ensure focal stimulation of the demyelinated area in a demyelinated axon model. We demonstrated, for the first time, that by activating the nodes in the demyelinated area, microcoil stimulation could rescue conductance failure in the locally demyelinated axons. These results suggest a potential novel intervention strategy for the treatment of local demyelinating conditions.

## 2. Methods

### 2.1. Multi-compartment, myelinated axon model

**Figure 1.**
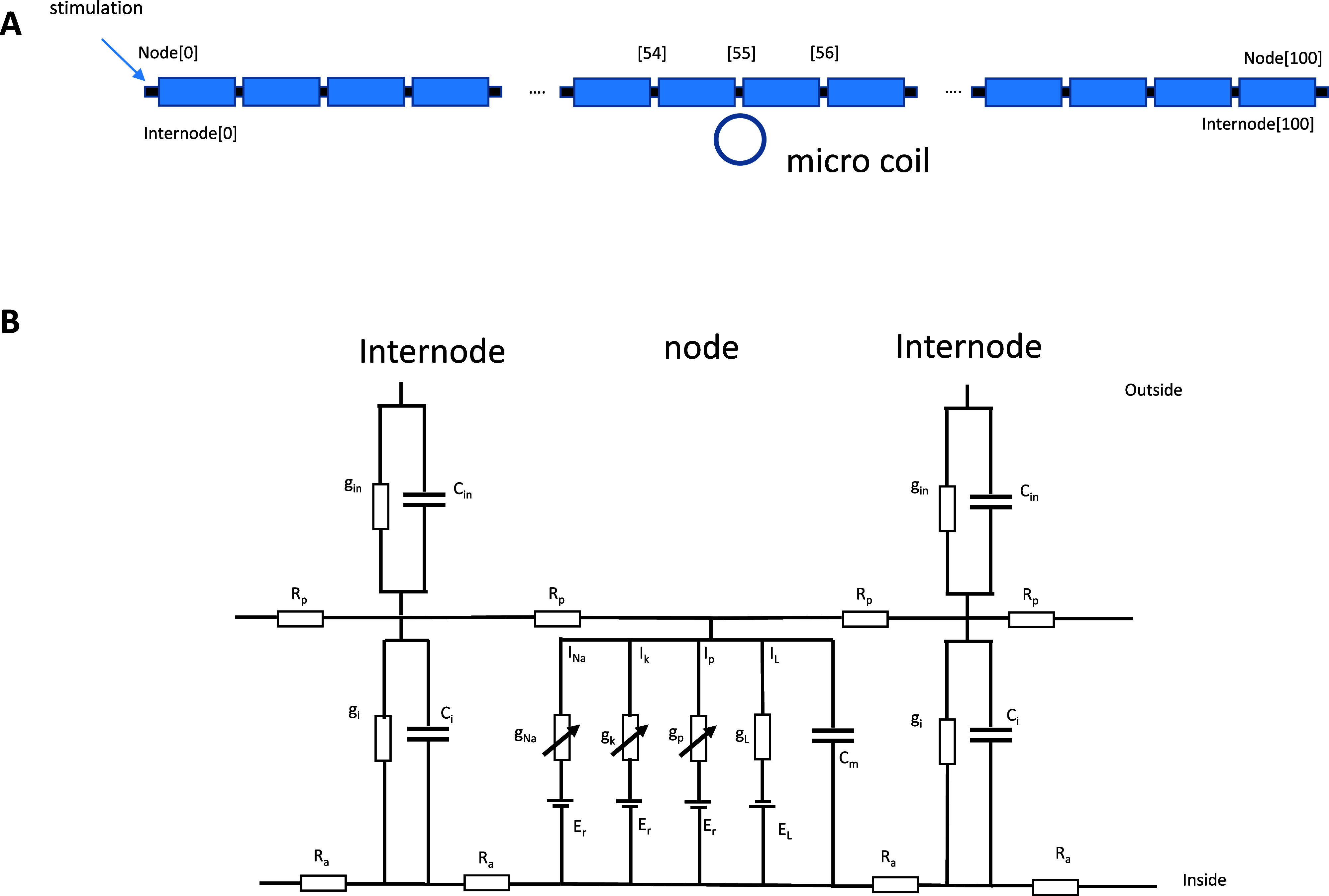
Multi-compartment model of a myelinated axon under microcoil stimulation. (A). The modeled axon contains 101 nodes and 100 internodes. (B). Each segment is characterized by its membrane conductance (node: *g*_L_; internode: *g*_i_) and membrane capacitance (node: *C*_m_; internode: *C*_i_). Node of Ranvier segments have additional voltage-dependent elements for sodium (Na), potassium (K), and non-specific (P) channels. Electrodynamics within each segment are expressed by relating axial, membrane leak, membrane capacitive, and ionic currents using Kirchhoff’s law (equation (A2)). The myelin sheath is represented by a linear conductance (*g*_in_) in parallel with capacitance *C*_in_. Longitudinally, the model contains cytoplasmic (*R*_a_) and periaxonal (*R*_a_) resistivities (see table 1).

The modeled myelinated axon was 100 000 *µ*m in length. It contained 101 nodes and 100 internodal segments. The internode length was 1000 *µ*m. The diameter of the entire fiber was 10 *µ*m and the axon diameter was 7 *µ*m (figure 1(A)). The nodes were described (appendix A, figure 1(B)) using the FH model. Equations and conductances that define the Na, K, and P channels at the node were given in (Frankenhaeuser and Huxley 1964), (Hines and Shrager 1991), and (Reilly 2016). The model parameters displayed in figure 1(B) are listed in table 1. The simulation code was modified from a myelinated model previously developed (Reilly 2016).

**Table 1.** Parameters of the modeled myelinated axon.

Parameters	Value	References
Fiber diameter (including myelin)	10 *µ*m	(Reilly 2016)
Number of nodes of Ranvier	101	(Reilly 2016)
Number of internodes	100	(Reilly 2016)
Width of node of Ranvier	2.5 e^−4^ cm	(Reilly 2016)
Axon diameter	7 *µ*m	(Reilly 2016)
Length of internode segments	1000 *µ*m	(Reilly 2016)
Total axon length	100 000 *µ*m	(Reilly 2016)
Cytoplasmic resistivity (*R*_a_)	100 ohm cm	(Reilly 2016)
Extracellular resistivity	300 ohm cm	(Reilly 2016), not used
Node membrane capacitance (*C*_m_)	2 *µ*F cm^−2^	(Frankenhaeuser and Huxley 1964, Hines and Carnevale 1997)
*P*_Na_	8 e^−3^ cm s^−1^	(Frankenhaeuser and Huxley 1964, Hines and Carnevale 1997)
*P*_K_	1.2 e^−3^ cm s^−1^	(Frankenhaeuser and Huxley 1964, Hines and Carnevale 1997)
*P*_P_	0.54 e^−3^ cm s^−1^	(Frankenhaeuser and Huxley 1964, Hines and Carnevale 1997)
*g*_L_	0.0303 S cm^−2^	(Frankenhaeuser and Huxley 1964, Hines and Carnevale 1997)
Reversal potential of leakage channel (*E*_L_)	−69.74 mV	(Reilly 2016)
[Na]_i_	13.74 mM	(Frankenhaeuser and Huxley 1964, Hines and Carnevale 1997)
[Na]_o_	114.5 mM	(Frankenhaeuser and Huxley 1964, Hines and Carnevale 1997)
[K]_i_	120 mM	(Frankenhaeuser and Huxley 1964, Hines and Carnevale 1997)
[K]_o_	2.5 mM	(Frankenhaeuser and Huxley 1964, Hines and Carnevale 1997)
Internodal capacitance (*C*_i_)	2 *µ*F cm^−2^ (single lamellae)	(Mcintyre *et al* 2002)
Internodal leak conductance (*g*_i_)	0.001 S cm^−2^ (single lamellae)	(Mcintyre *et al* 2002)^a^
Myelin capacitance (*C*_i_)	0.1 *µ*F cm^−2^	(Mcintyre *et al* 2002)
Myelin conductivity (*g*_i_)	0.001 S cm^−2^	(Mcintyre *et al* 2002)
Resting potential (*E*_r_)	−70 mV	(Reilly 2016)
Temperature	20 Celsius	(Reilly 2016)

^a^ -STIN conductance in the MRG model (Mcintyre *et al* 2002).

To adapt the model to simulate pathological alterations in myelinated axons, we implemented a modified version of the core conductor model of myelinated axons (Koles and Rasminsky 1972, Basser 2004, Resnick *et al* 2018). The core model treated the internodal segment of the myelinated axons as a single layer of resistive–capacitive medium separating resistive compartments. We also assumed that demyelination did not affect the nodal membrane, physiological properties of the node, or the axon diameter, as in (Koles and Rasminsky 1972).

The myelin sheath is formed by the repeated wrapping of a glial cell membrane around the axon, creating a multi-layered structure that insulates the axon and allows for rapid signal transmission. Demyelination causes the reduction of myelin thickness while increasing the conductance (*g*_in_) and capacitance (*c*_in_) of the myelin sheath (Koles and Rasminsky 1972, Mcintyre and Grill 1999, Mcintyre *et al* 2002, Resnick *et al* 2018). Numerical increase of the myelin conductance and capacitance can be used to simulate demyelination process (Sleutjes *et al* 2019). Others have linked myelin thickness with changes in myelin conductance and capacitance. For example, in the MRG model, the lamella membrane conductance (*g*_i_ = 1 × 10^−3^ S cm^−2^) and lamella membrane capacitance (*c*_i_ = 0.1 *μ*F cm^−2^) (Mcintyre *et al* 2002) were divided by the number of lamella to produce overall myelin conductance and capacitance (Mcintyre *et al* 2002). In our simulation, since individual layers were not modeled, we scaled the overall myelin conductance and capacitance with changes of the myelin thickness, as reported previously (Koles and Rasminsky 1972, Resnick *et al* 2018), using the following equations,

\begin{align*}{g_{{\text{in}}}}\left( {d\% } \right) = {g_{\text{i}}}/d\end{align*}

\begin{align*}{c_{{\text{in}}}}\left( {d\% } \right) = {c_{\text{i}}}/d\end{align*}

where *g*_in_*(d%)* represents the overall myelin conductance and *c*_in_*(d%)* the overall myelin capacitance when the myelin thickness was reduced to *d*%, respectively.

A shortcoming of this approach is that the myelin conductance and capacitance approach infinity in the case of complete myelin loss. As in (Resnick *et al* 2018), we address this problem by incorporating the internodal plasma membrane conductance and capacitance in series with the myelin sheath’s, creating a core-dual conductor model (figure 1(B)). The total internodal conductance and capacitance can be calculated by adding plasma and myelin contributions in series. This procedure was implemented in all subsets of internodes, allowing us to simulate any forms of partial and complete demyelination of any internodes.

### 2.2. The microcoil model that generates focal stimulation

**Figure 2.**
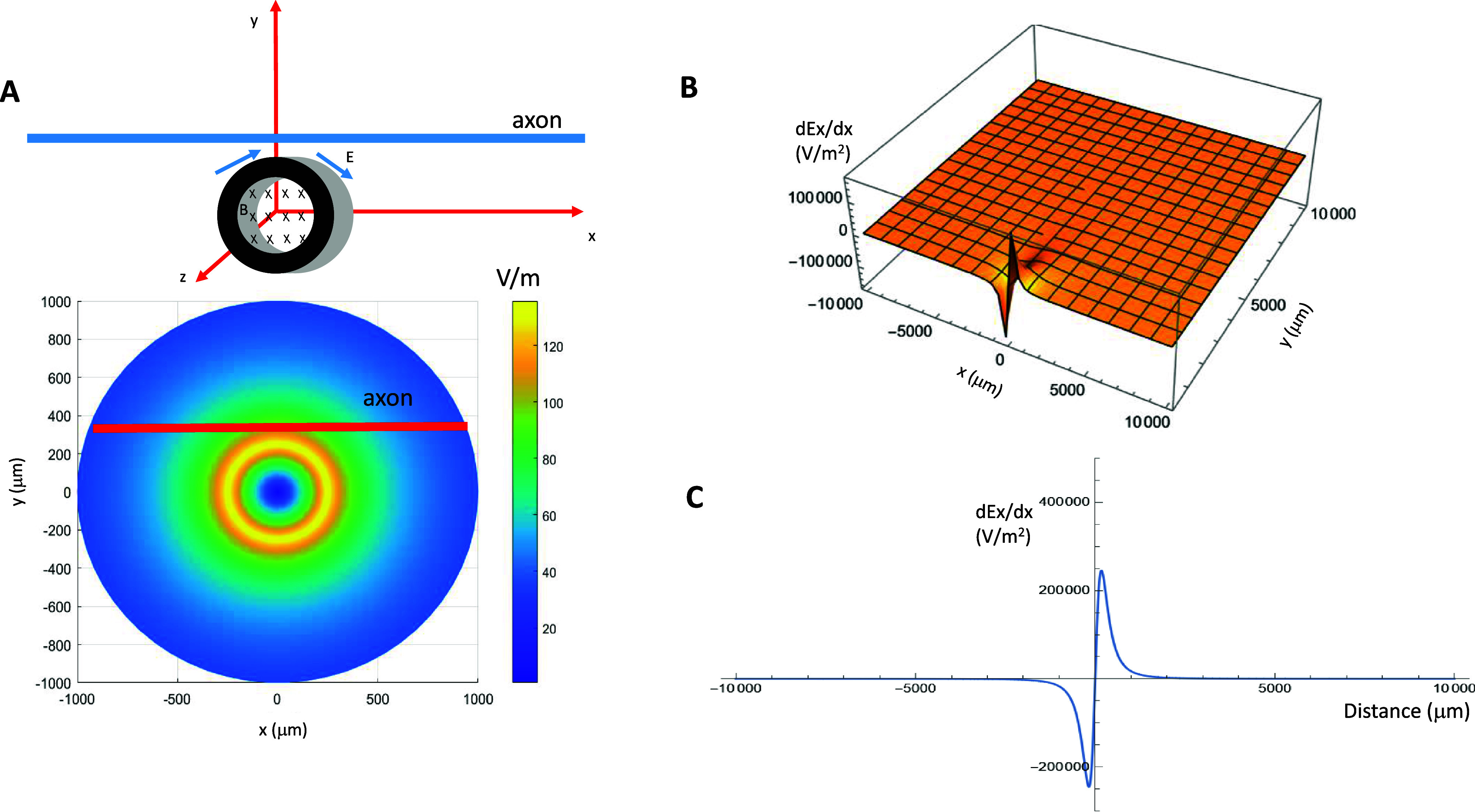
Distribution of induced electric field generated by a microcoil and its gradient along the axon. (A). Electric field distribution around the axon. The coil is located at the center of the axon, 50 *µ*m away from the axon. (B): magnetic field; *E*: induced electric field. *B*. Gradient of the induced electric field in the axon direction (*x*-direction) and its dependence on the coil-axon distance (*y*). (C). Electric field gradient along the axon.

The microcoil model was developed in our previous work (Ye 2022) and was used to stimulate the demyelinated axon (appendix B). Briefly, we modeled the submillimeter microcoil as a multiple-loop circular structure (figure 2(A)). The coil parameters are listed in table 2. These parameters closely match the miniature coils that were used in our experiments to stimulate neurons from invertebrates (Skach *et al* 2020, Ye *et al* 2022a, 2024) and rodents (Ye *et al* 2020).

**Table 2.** Coil parameters.

Parameter	Value	References
Inductance (*L*)	100 nH	(Skach *et al* 2020)
Maximal current (*I*_max_)	200 mA	(Skach *et al* 2020)
Resistance (*R*)	2 Ω	(Skach *et al* 2020)
Coil length (*l*)	0.5 mm	(Skach *et al* 2020)
Radius (*R*_c_)	0.25 mm	(Skach *et al* 2020)
Number of loops (*N*)	20	(Skach *et al* 2020)

*Orientation of the coil:* Several recent experiments have suggested that microcoil orientation played a pivotal role in axonal stimulation (Golestanirad *et al* 2018, Saha *et al* 2022a). In this work, the coil was positioned to induce an electric field parallel to the axons to generate effective stimulation (Jefferys 1981, Gluckman *et al* 1996). During the simulation, the distance between the coil and the axon was equal to or greater than 50 *µ*m (thickness of the biocompatible coating of the coil during experiment).

*Spatial profile of the stimulation:* Previously, we have calculated the intensity of the electric field induced by the coil ((Ye 2022), also see appendix B), and found that the small size of the coil ensured that a relatively large electric field was generated locally around the coil. Furthermore, we calculated the gradients of the induced electric field along the axon (figures 2(B) and (C)) and used this data to provide the most effective activation of the node of interest.

**Figure 3.**
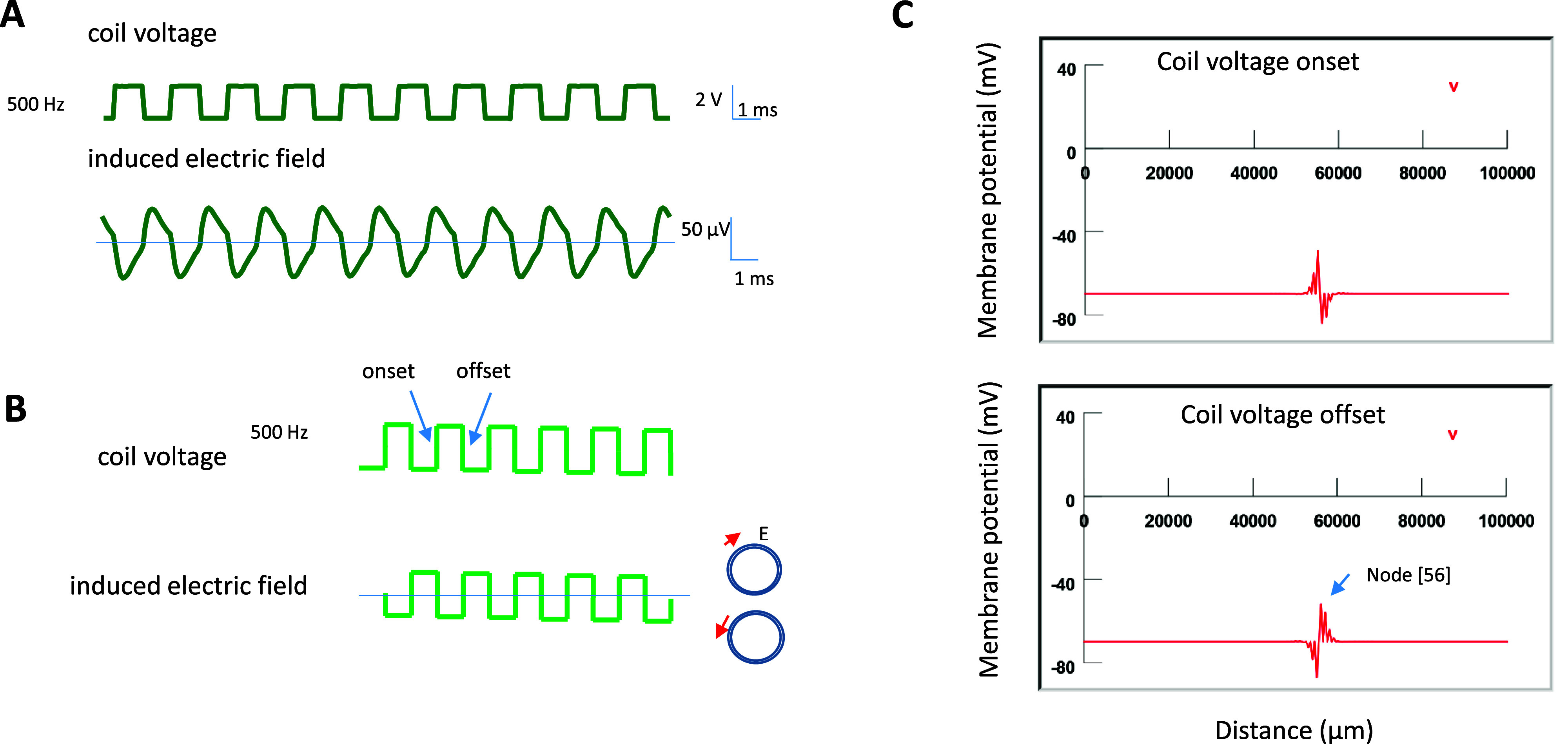
Magnetic stimulation with the microcoil causes changes in local node membrane potential. (A). Voltage pulses (500 Hz, 1 ms pulse width) in the coil induce an electric field around the coil (experimentally measured). Electromagnetic induction happens at the rising and falling phases of the voltage pulses, producing a biphasic electric field. (B). In NEURON simulation, the biphasic waveform is programmed and applied to the myelinated axon model for stimulation. Red arrows: direction of the induced electric field around the coil. Note the polarity of the field corresponds with the direction of the induced electric field (*E*). (C). Membrane potential in several nodes during a subthreshold stimulation by the microcoil (NEURON simulation). Node [56] is targeted and its membrane potential change is most obvious. The membrane potential is generated at the onset (top panel) and offset (bottom panel) of the coil current, which induced electric field along the myelinated axon. The membrane potential in this area oscillates at the frequency of the coil current during stimulation.

*Temporal profile of the magnetically induced electric field:* When a current pulse was delivered into the coil for neural stimulation, it induced the electric field at the onset and offset of the pulse, as confirmed by our calculation (appendix B). The induced electric field was biphasic in shape (figure 3(A)), as previously confirmed by our group (Skach *et al* 2020, Ye and Barrett 2021) and others (Minusa *et al* 2018) under various stimulation conditions, such as dorsal cochlear nucleus (Golestanirad *et al* 2018) and cortical neuron (Lee and Fried 2017) stimulation. In the model, we displayed the induced electric field as biphasic pulses (figure 3(B)).

### 2.3. Model microcoil stimulation of a demyelinated axon

The multi-compartment, myelinated axon model and the microcoil model were implemented together using the NEURON (v7.8) simulation environment package (Hines and Carnevale 1997). Simulations used NEURON’s built-in adaptive integrator with DASPK, a differential algebraic solver with preconditioned Krylov method (Brown *et al* 1994). The model was operated at room temperature (20 °C).

To study the effect of demyelination on axonal conductance, a physiological AP was simulated by activating the node 0 with a pulse (2 nA in amplitude and 0.1 ms in pulse width). Timing of the AP to invade the demyelinated area was adjusted by altering the delay of the injected pulse. Local demyelination was simulated by reducing the myelin thickness to different levels, and the deficit in axonal conductance was characterized by measuring the delay or capability for the AP to travel through the demyelinated area.

To study the capability of the microcoil stimulation in recovering/reinstalling axonal conductance in the demyelinated axon, biphasic short pulses in alternating directions were utilized to simulate the waveform of the coil-induced electric field in NEURON (figure 3(B)). The extracellular stimulus by the coil was implemented directly, i.e. by controlling the potential at the outer surface of each model compartment. This potential is calculated by integrating the scalar component of the electric field along the axon path (appendix B, equations (B8) and (B9)). Microcoil stimulation caused oscillation of local membrane potential (figure 3(C)), whose profile matches perfectly with the gradient of the electric field (figure 2(C)).

During the simulation, the program first searched for a threshold for AP initiation by the coil stimulation alone. Threshold of axonal activation was identified as the lowest stimulus intensity that could initiate an AP in the myelinated axon. We then adjusted the stimulation intensity for subthreshold stimulation. The intensity for subthreshold stimulation was normalized to this identified threshold.

Internode segment length, diameter, and axonal resistance remained constant for all described experiments, while myelin thickness, capacitance, and resistance were experimentally manipulated. The impact of the coil on various locations of the demyelinated axons was tested, guided by the analysis of the induced electric field and gradient (appendix B). Two frequencies (100 Hz and −5000 Hz) were tested and compared to investigate the frequency-dependent effects. To demonstrate the underlying ion channel responses to the microcoil stimulation during conductance restoration, membrane currents and state variables or the targeted nodes in the demyelinated area were analyzed.

## 3. Results

To investigate the possibility of rescuing axonal conductance failure after demyelination with electromagnetic stimulation, we built a multi-compartment NEURON model of a demyelinated axon under focal electromagnetic stimulation with a miniature coil. We computed the electric field generated by the coil and applied it to the axonal model. We simulated AP propagation in the axon when local demyelination occurred. We positioned the coil next to the demyelinated area to activate nodes in the demyelinated region. We analyzed the impact of coil location, stimulus frequency and degree of demyelination on the success of functional rescuing of the axon conductance. We also investigated ion channel dynamics during magnetic stimulation and recovery of axonal function.

### 3.1. Microcoil stimulation depolarizes node membrane potential, and the location of depolarization is predicable by the gradient of the magnetically induced electric field

Under time varying magnetic stimulation, neural tissue is stimulated by the magnetically induced electric field via magneto-electric induction (Ye *et al* 2011, 2022b). Due to its miniature size, the microcoil can generate a very focal electric field distribution around the coil, to provide local activation of the subcellular structure (Bonmassar *et al* 2012, Lee *et al* 2016, Ye and Barrett 2021). Previously, we have computed the induced electric field distribution generated by a circular miniature coil (Ye 2022), and have validated the effects of the focal stimulation using various preparations, such as mouse hippocampal neurons (Ye *et al* 2020) and invertebrate ganglion cells (Ye and Barrett 2021, Ye *et al* 2022a, 2024). This coil has a diameter of 0.5 mm, and contains 20 loops of circular wires (table 2), representing a commercially available coil used for neural activation in our experiments (Ye *et al* 2020, 2024, Ye and Barrett 2021). Therefore, the parameters that describe the microcoil closely match those used in the experiments.

In our model, we positioned the microcoil close to the myelinated axon (assuming 50 *µ*m thickness of the coated material, figure 1(A)) and computed the magnetically induced electric field. The node contained F–H ion channel mechanisms (figure 1(B), appendix A). Figure 2(A) demonstrates the spatial distribution of the induced electric field around the myelinated axon. The axon could experience a local electric field intensity as large as 50–80 V m^−1^ (figure 2(A)). Previously, a 10 V m^−1^ threshold was reported for neuronal activation (Chan and Nicholson 1986). Fields at 10–20 V m^−1^ are sufficient to modulate neuron firing in Purkinje and stellate cells *in vitro* (Chan and Nicholson 1986), or in the guinea pig hippocampus (Jefferys 1981).

As the miniature size coil allows subcellular stimulation, finding the location of neural activation is critical to target the fine structure, such as a node on the axon. Previous studies have established that the gradients of the electric field along the axon define the location and speed of depolarization or hyperpolarization by the extracellular stimulation (Rattay 1986, Lee and Fried 2017). Figure 2(B) plots the field gradient (d*Ex/*d*x*) along the axon, and its dependency on the coil-axon distance (*y*). When the coil is located 50 *µ*m away from the axon, it can produce a field gradient as large as 250 000 V m^−2^ (figure 2(C)), well above the value that can cause neural activation (50 000 V m^−2^ in (Lee *et al* 2016)). As the coil moves away from the axon, d*Ex/*d*x* decreases, suggesting a decreased field gradient and decreased efficacy of stimulation.

Previous work has theoretically (Ye 2022) and experimentally (Bonmassar *et al* 2012) confirmed that the rising and decaying phases of a coil current can lead to a large change in the magnetic field and the induced electric field (figure 3(A)). Therefore, in this study, we modeled the induced electric field using biphasic voltage (figure 3(B)) and applied it to locally demyelinated axons. To confirm that the field gradient indeed predicts the location of membrane polarization, we applied a subthreshold stimulus to the NEURON model (to avoid triggering of the AP). The coil stimulation depolarized several nodes next to the coil (figure 3(C)), and the amount of depolarization in each node is defined by the profile of the field gradient (figure 2(C)). Because the induced electric field is biphasic, we observed a depolarization/hyperpolarization oscillation in the membrane potential (figure 3(C)). Therefore, it is possible to position the coil along the axon to selectively target a specific node for stimulation with various frequencies.

### 3.2. Demyelination causes delay and blockage of AP, depending on the severity of demyelination

**Figure 4.**
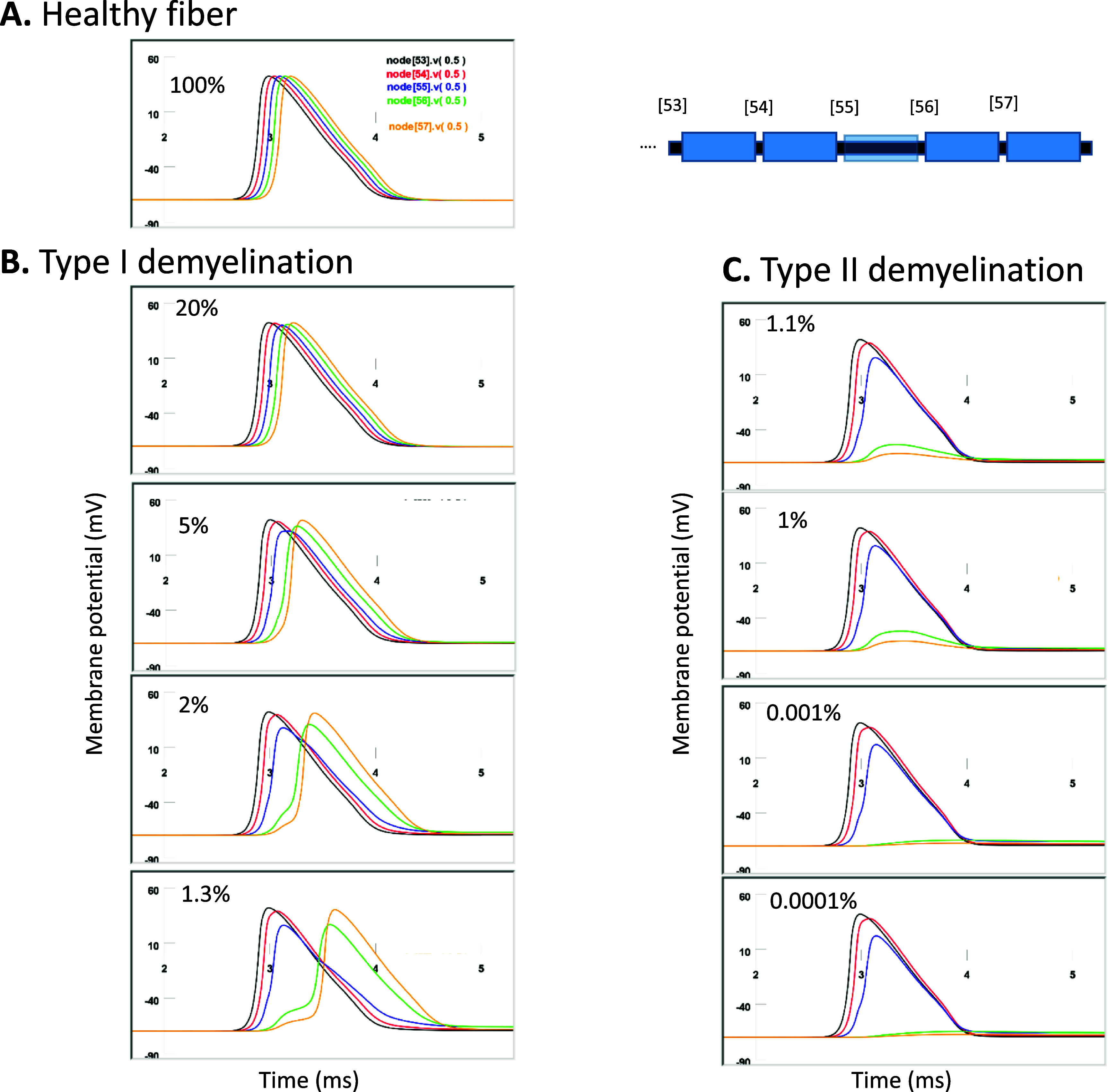
Demyelination causes failure of axonal conductance. Action potential is initiated at node 0. Illustrated here is the propagation of an action potential in the simulated axon at nodes 53–57. Node [53].v(0.5): membrane potential in node 53. (A). normal fiber with 100% myelin thickness. (B). Type I demyelination. Myelin of internode 55 is reduced to 20%, 10%, 5%, 2%, and 1.3% original thickness. Decreased myelin thickness causes prolonged delay for AP propagation. (C). Type II demyelination. Myelin of internode 55 is significantly reduced (below 1.1% original myelin thickness). Type II axon demonstrates complete failure of axonal conductance.

When simulating AP propagation in the modeled axon, we injected an intracellular current to node 0 to initiate an AP. In the healthy, myelinated axon, the AP propagated to the other side of the axon at a constant speed 22 m s^−1^ (supplementary figure 1), confirming the results from previous work (Reilly 2016). To monitor the behavior of the nodes during AP propagation, we plotted the membrane potentials in nodes 53–57 (figure 4(A)). In the subsequent experiments, the microcoil was positioned close to these nodes for magnetic stimulation. Among these nodes, node 56 was designated as the ‘targeted node’ by coil stimulation, whose ion channel dynamics will be investigated in greater detail.

To simulate local demyelination, the capacitance and resistance of the myelin are linearly scaled by the loss of myelin thickness (Mcintyre *et al* 2002). Figure 4 illustrated the AP traveling in nodes 53–57 when internode 55 (the internode between nodes 55 and 56) was uniformly demyelinated from 100% thickness to complete loss of the myelin sheath. Decrease in the myelin thickness below 10% led to a significant delay between the APs in nodes 55 and 56. We name these axons as *Type I demyelinated axons* as they carry the APs with a certain delay (figure 4(B)). The fiber failed completely to conduct the nerve pulse when the myelin thickness fell below 1.3% (figure 4(C) and supplementary figure 2). We name these axons as *Type II demyelinated axons*.

### 3.3. Microcoil stimulation rescues axonal conductance in the demyelinated axon

**Figure 5.**
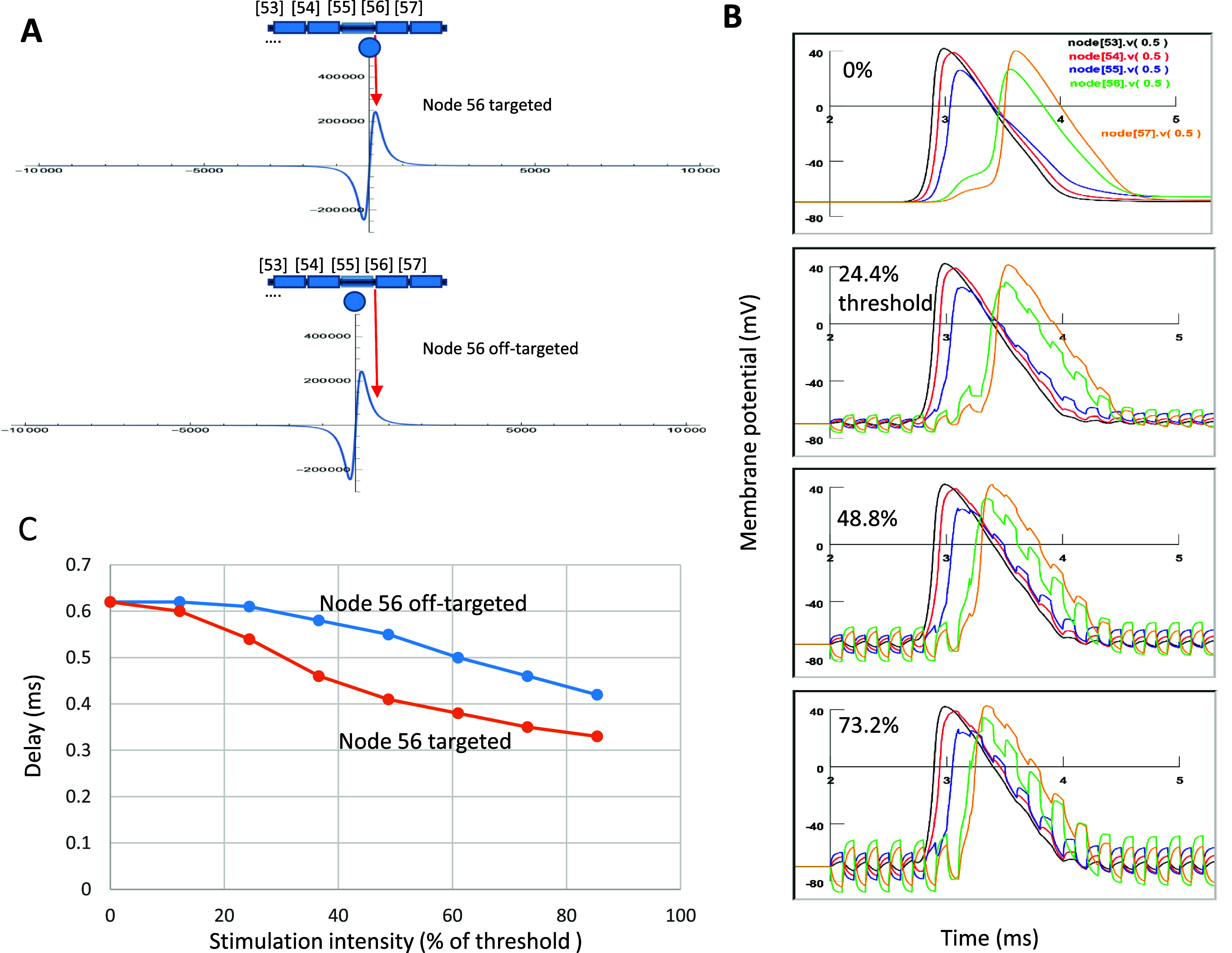
Microcoil stimulation shortens the delayed action potential propagation in the Type I demyelinated axons. (A). Orientation of coil to the targeted node 56 in the demyelinated axon. Top: Node 56 is targeted and stimulated by the electric field with maximal field gradients. Bottom: Location of the coil in the middle of the demyelinated internodal 55, resulting in roughly one fourth of field gradient applied to node 56. (B). Action potentials recorded between nodes 53–57. Significant reduction in myelin thickness (to 1.3%) causes a significant delay of AP. When node 56 is targeted, coil stimulation shortened the delay between nodes 53–57 in an intensity-dependent manner. Increase in stimulating intensity shortens the conductance delay. (C). Node-targeted stimulation is more effective in shortening the delay than node off-targeted stimulation.

To study the effects of microcoil stimulation on axonal conductance in locally demyelinated axons, we position the microcoil so that the location of maximal field gradient overlapped with the location of node 56 (figure 5(A), top panel). This allowed the microcoil to apply optimal stimulation to the targeted node.

Figure 5(B) demonstrates the effects when coil stimulation was on a *Type I demyelinated axon*. A significant delay of AP traveling between nodes 53–57 occurred when the myelin thickness between nodes 55 and node 56 was reduced to 1.3%. A 5000 Hz magnetic stimulation shortened this delay. Effects of coil stimulation depended on its intensity: stronger stimulation resulted in further reduction of the delay (figure 5(C)).

To investigate the impact of coil location on the success of shortening this delay, we relocated the microcoil to the middle of the demyelinated region (internode 55), and compared it with the case when the coil directly targeted the node 56. At this location, the amplitude of the field gradient was about one fourth of its maximal on node 56 (figure 5(A), bottom panel). At the off-target location, the microcoil stimulation could still shorten the conductance delay in an intensity-dependent manner. However, the same amount of stimulation was far less effective when compared with the case of direct, close stimulation on the targeted node 56 (figure 5(C)).

**Figure 6.**
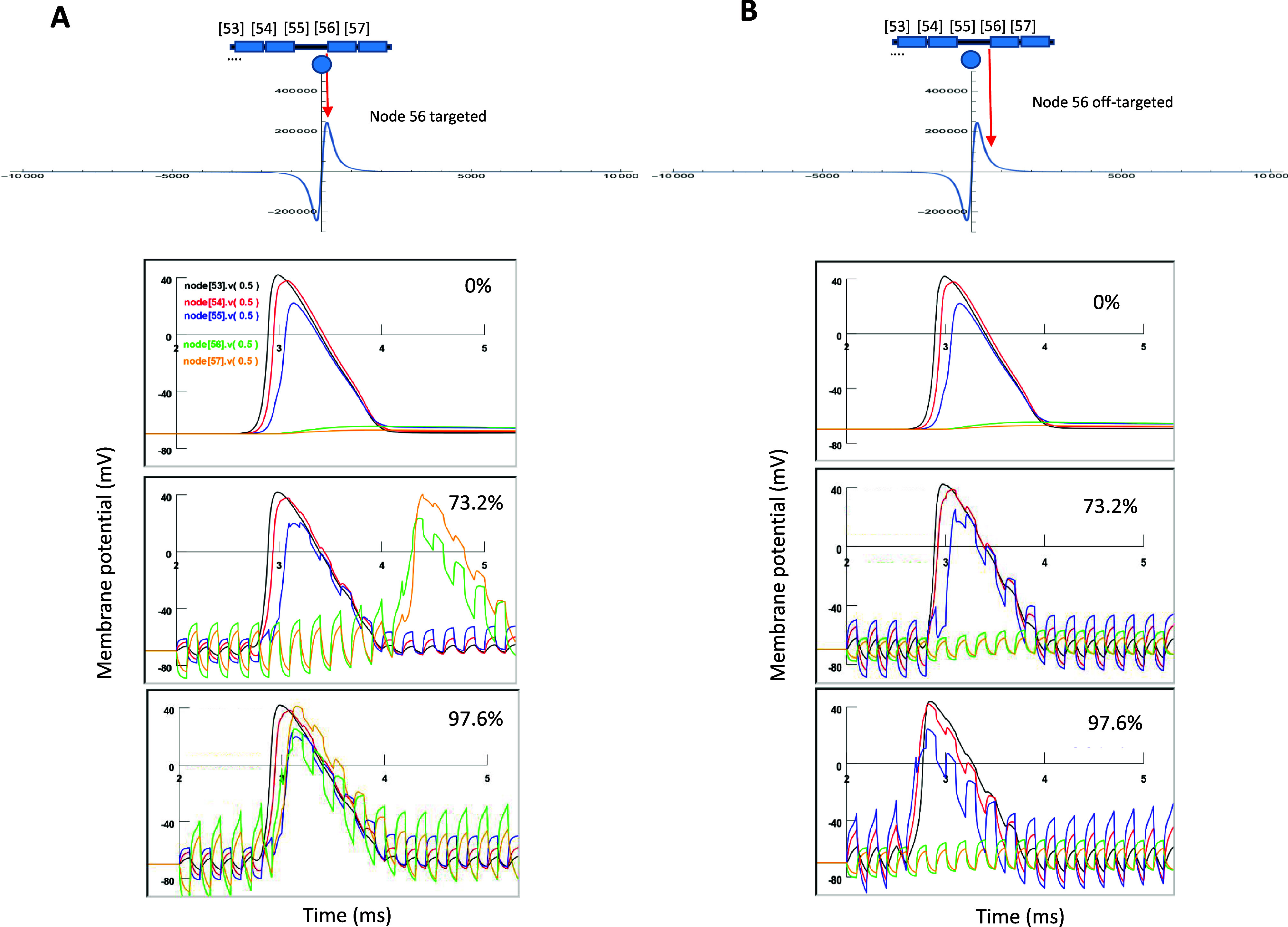
Microcoil stimulation rescues axon conductance in a completely demyelinated axon (Type II). (A). Orientation of coil to the targeted node 56 in the demyelinated axon. Node 56 is aligned with the peak of the electric field gradient for optimal stimulation. Complete demyelination between node 55 and 56 causes a complete axonal blockage. Stimulation intensity is increased from 0% to 97.6% of threshold, which almost completely rescued the axonal conductance (compare to AP propagation in the healthy axon in figure 4(A)). (B). Coil stimulation with poor location is insufficient to restore the axonal conductance. The coil is positioned in the middle section of internodal 55, with the maximal field gradient off-target from node 56. Maximal subthreshold stimulation cannot restore the action potential propagation from nodes 55–56.

Figure 6 demonstrates the effects when coil stimulation was on a *Type II demyelinated axon*, in which complete axonal blockage occurred. When the coil stimulation was targeted on node 56 (figure 6(A), top panel), interestingly, we observed that coil stimulation could enable the AP to travel through the demyelinated region when the stimulation was above 61.0% of threshold amplitude. At this moderate stimulation intensity, we observed a jerking/delay when the AP traveled through the demyelinated area and a distortion of the overall potential profile (supplementary figure 3). Further increases in the stimulation intensity could further shorten this delay. At 97.6% of threshold intensity, the AP could travel through the demyelinated area without noticeable delay, reminiscent of a healthy, myelinated axon (figure 4(A)).

To investigate the impact of coil location on the success of restoring axonal conductance in the Type II demyelinated axon, we relocated the microcoil to the middle of the demyelinated region (internode 55, figure 6(B)), and compared it with the case when the coil directly targeted node 56 (figure 6(A)). Here, the microcoil stimulation was incapable of resuming axonal conductance in the Type II demyelinated axon. These results indicate the importance of direct targeting of the node for rescuing the axons with complete conductance failure.

Although we were able to establish that microcoil stimulation could restore axonal conductance in demyelinated axons, the effects of microcoil stimulation on axonal conductance in healthy, myelinated axons require further investigation. As in the case of demyelinated axons, microcoil stimulation caused local membrane potential oscillations in the healthy axon. However, the subthreshold oscillation of the membrane potential caused by coil stimulation did not affect the AP propagation in the healthy axon. There was no obvious delay or acceleration in the AP as it traveled through the oscillating zone (supplementary figure 4).

### 3.4. Role of stimulation frequency in the restoration of axonal conductance in the demyelinated axon

Data demonstrated so far indicates that that axonal conductance recovery by microcoil stimulation is a consequence of the synergic effects of the invading AP and the membrane depolarization in the microcoil-targeted node. Since membrane potential oscillation is directly controlled by the stimulation frequency, we hypothesize that relatively high frequency stimulation and fast oscillation in the membrane potential is necessary to restore axonal conductance in the severely demyelinated axon.

**Figure 7.**
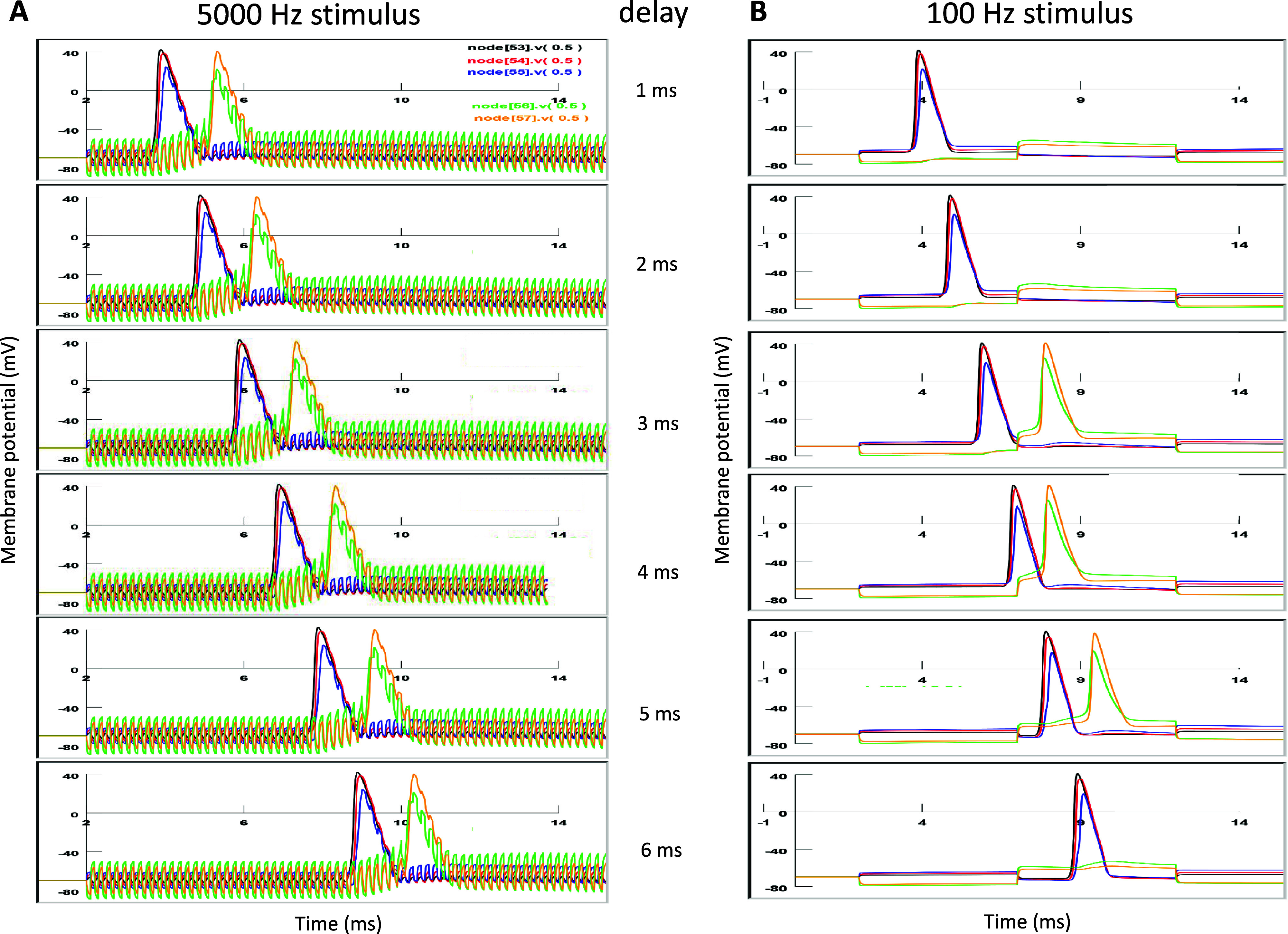
Conductance restoration by microcoil stimulation is dependent on the stimulus frequency. Location of the coil is at the optimal site and node 56 is targeted. Action potential triggered in node 0 is assigned with variable delay (1–6 ms) to simulate the variable timing of the invading action potential to the demyelinated area. (A). 5000 Hz stimulation can restore the conductance, regardless of the invading time of the action potential to the demyelinated area. (B). 100 Hz is not reliable in rescuing the axonal conductance if the invading action potential does not temporally match well (i.e. when the delay is 1 ms, 2 ms, and 6 ms) with the membrane potential oscillation in the targeted node 56.

To test this hypothesis, we compared the effects of 100 Hz and 5000 Hz stimulation in the Type II, severely demyelinated axon (local demyelination between nodes 55 and 56). To test the liability of coil stimulation on the arbitrary invading APs, we initiated APs in node 0 with variable delay times. Figure 7(A) demonstrates the effect of 5000 Hz stimulation. The fast depolarization/hyperpolarization on the membrane potential in node 56 prepared the node for the invading AP. Therefore, timing of arrival of the invading AP was not important, and node 56 could be activated by the combined effects of invading AP and the instant depolarization of the node by the coil stimulation. In contrast, figure 7(B) demonstrates that 100 Hz stimulation could not provide the reliable assistance needed to propagate the AP. We adjusted the invading time of the AP, and found that only when the invading AP has a temporal match with the membrane potential oscillation could it travel through the demyelinated area. Therefore, high frequency stimulation, by preparing the node membrane potential, is essential for the invading AP traveling through the demyelinated region.

### 3.5. Mechanisms of conductance restoration by microcoil stimulation in the demyelinated axon

To delineate the biophysics mechanisms underlying the effects of microcoil stimulation in restoring axonal conductance, we examined the interaction between the microcoil-induced electric field and the nodes/internodes in the demyelinated area. Specifically, demyelination occurred in internodal 55, leading to complete axonal conductance failure, and node 56 was targeted by the micro-coil (figure 6(A)). Our analysis focused on the membrane potential, currents, and ion channel dynamics in the three representative nodes, 55, 56, and 57, and leakage currents in internode 55. These components were the most relevant structures involved in the demyelination and function restoration processes.

#### 3.5.1. Restoration of axonal conductance by microcoil stimulation is mediated by the enhanced node current in the demyelinated region

**Figure 8.**
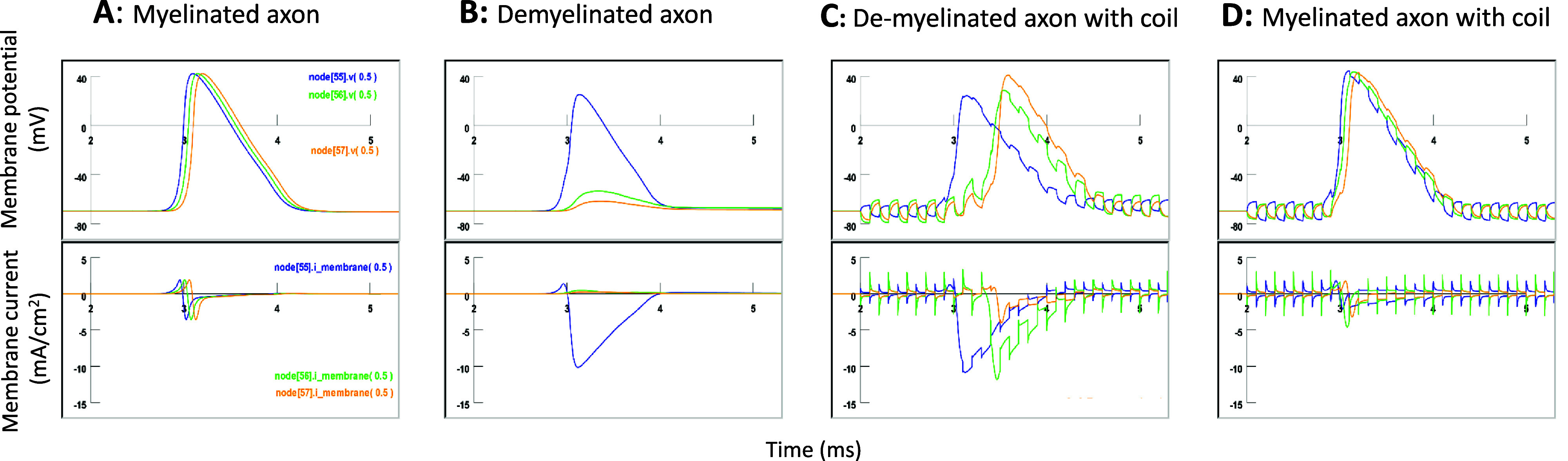
Micro coil stimulation restores node currents in the demyelinated area. Plotted here are membrane potentials and currents in three representative nodes (55, 56, and 57) when an action potential travels through them. The invading action potential is initiated by intracellular stimulation of node 0. (A). Healthy, myelinated axon without microcoil stimulation. (B). Axon demyelinated and conductance blockage between nodes 55 and 56 (Type II demyelination). The action potential reaches node 55 but fails to reach node 56. Node 56 and node 57 receive small outward current, insufficient to initiate an action potential. (C). Axonal conductance is restored by the microcoil stimulation. Activation of node 56 is mediated by a large inward current. (D). Healthy, myelinated axon under microcoil stimulation. The coil-induced membrane oscillation has minimal effect on AP propagation and the underlying membrane current.

In a healthy, myelinated axon, nodes 55–57 were sequentially activated by the invading nerve pulse (figure 8(A)). The total membrane current in these nodes was characterized by an outward current, followed by an inward current (figure 8(A), bottom). The early outward membrane current in a specific node was mainly mediated by the activation of the upstream node. For example, when node 55 was activated, a local current generated by the AP in node 55 passively flowed down the axon through the internode 55. This axial current eventually flowed out of the membrane of the downstream node 56, depolarizing the membrane for AP initiation, and thereby advancing the AP.

When demyelination occurred between nodes 55 and 56 (figure 8(B)), the invading AP traveled to node 55. Node 55 could still generate a relatively small AP, even though the neighboring internode 55 became electrically leaky. Interestingly, because the membrane conductance of internode 55 was significantly increased, the depolarization of the membrane in node 55 drove a much larger inward current that leaked out of internode 55. Unfortunately, this current could not generate a sufficient axial current inside the leaky internode 55. The initial outward membrane current in the downstream node 56 was insufficient to reach the activation threshold. Consequently, the initiation and sustenance of the AP is hindered, resulting in conductance failure.

When the axon was under microcoil stimulation, oscillation in the membrane potential played a pivotal role in the restoration of axonal conductance in the presence of the invading AP (figure 8(C)). At node 55, the AP was triggered by a sufficient early outward membrane current. In the targeted node 56, early depolarization of the membrane by the outward membrane current (driven by the AP in the upstream node 55) was weak in the absence of coil stimulation. However, when magnetic stimulation was applied to this node, membrane depolarization at node 56 was boosted. Early membrane depolarization in node 55 was largely due to the combined effects of coil-induced membrane oscillation and AP-driven depolarization. This enhanced depolarization provided an opportunity for the membrane potential in node 56 to pass the threshold for activation. When the AP was resumed at node 56, the early outward membrane current was sufficiently strong in the downstream node 57, leading to AP initiation at this node.

For the healthy, myelinated axon, microcoil stimulation and its associated membrane potential changes did not affect the axonal conductance (figure 8(D), supplementary figure 4). Membrane potential oscillations in nodes 55–57 did not alter the AP initiation sequence. Sufficient early currents were observed at all three nodes for AP initiation.

#### 3.5.2. Restoration of axonal conductance by microcoil stimulation is mediated by the recovery of normal channel dynamics in the demyelinated area

To further investigate the ion channel dynamics underlying the restored axonal conductance by microcoil stimulation, we plotted the membrane potential, individual channel currents, and channel state variables in microcoil-targeted node 56.

**Figure 9.**
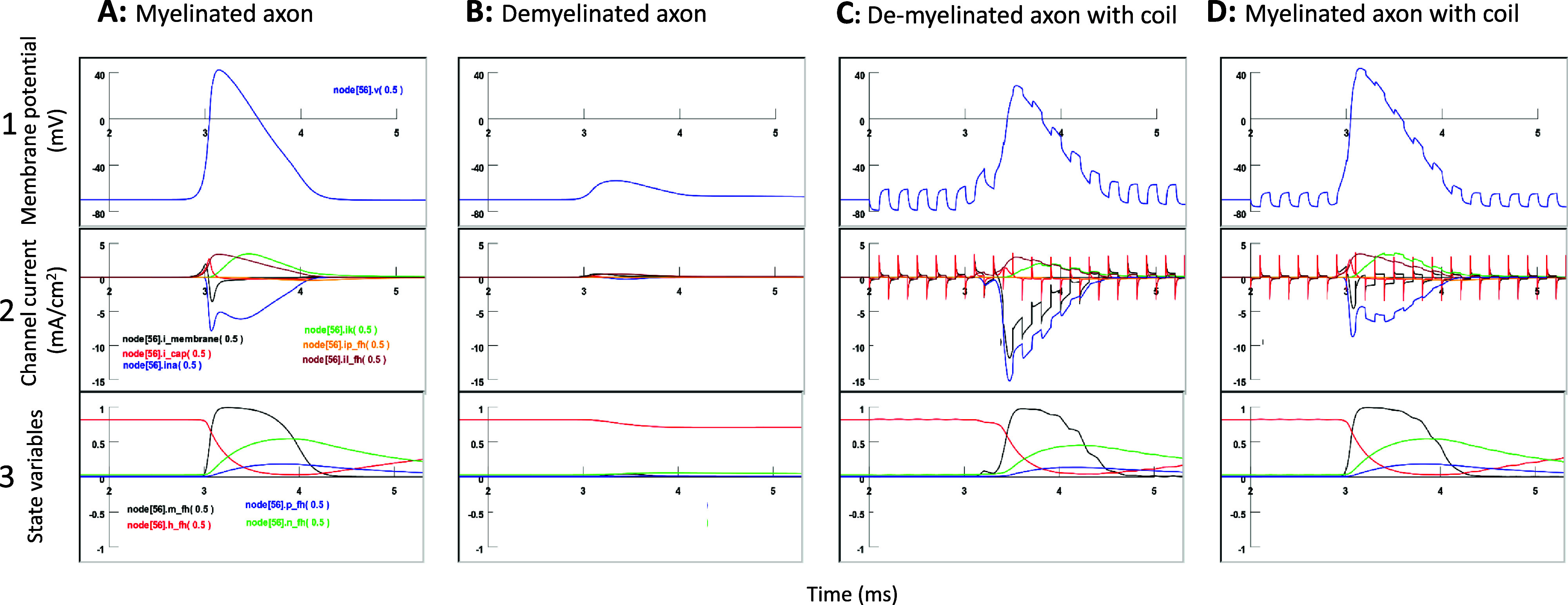
Micro coil stimulation restores channel dynamics in the demyelinated area. Membrane potential (1), individual channel current (2), and channel state-variables (3) that mediate the action potential in the targeted node [56]. (A). A healthy, myelinated axon without microcoil stimulation. (B). Axon demyelinated (Type II) between nodes 55 and 56. Insufficient activation (*m*) in the sodium channel (*m* variable) is observed using complete conductance blockage. (C). The demyelinated axon in (B) was rescued by the microcoil stimulation on the targeted node 56. After node 56 resumes its activation, its ion channel dynamics resemble those of the myelinated axon (A3). (D). A healthy, myelinated axon under microcoil stimulation. Although coil stimulation causes membrane potential oscillation, the ion channel dynamics resemble those without magnetic stimulation (A3).

In the healthy axon (figure 9(A)), when the AP traveled through node 55, several ion currents flowed across the membrane. This included a large, fast, inward Na^+^ current (iNa), a relatively fast, non-specific, delayed current (ip_fh), a slow outward K^+^ current (iK), and leakage channel current (il_fh) defined in the F–H model. At this moment, the membrane current (i_membrane) was equivalent to the sum of the capacity current (i_cap) and all the channel currents (equation (A2)). During the AP, i_membrane became inward, primarily due to the large inward iNa. A large portion of this inward current flowed through the axis to the downstream node 56 and became the outward membrane current for membrane depolarization. i_membrane became zero (balanced by the large inward iNa and outward iK and il_fh) shortly after the peak AP. The large inward current was mediated by the fast activation of the sodium channel, whose channel dynamics can be described by *m*^2^*h* (Frankenhaeuser and Huxley 1964). In fact, we observed an abrupt increase in the activating variable (*m*) and relatively slow decrease in the inactivating variable (*h*). Activation of the potassium and non-specific channels was relatively slower than that of the Na channel, as demonstrated by the activating variable (*n*) for the potassium channel and activating variable (*p*) for the non-specific channel, respectively.

Figure 9(B) demonstrates the case of demyelination and conductance failure between node 55 and node 56. Node 56 demonstrated weak depolarization, as well as weak sodium, potassium, and non-specific delayed currents. By further exploring the state variables in node 56, we found that the modest depolarization caused by the weak axial current could only lead to a slight increase in the *m* and *n* variables and a slight decrease in the *h* variable, indicating that neither the Na nor K channels were fully activated.

Figure 9(C) illustrates the case when axonal conductance was restored by the microcoil stimulation (figure 6(A)). When the AP is finally triggered in node 56, an enhanced net membrane current was observed in the node. This enlarged net membrane current was due to enhanced Na channel current. The increased Na current/K current ratio keeps node 56 activated, ensuring the propagation of the AP through the demyelinated area. When node 56 was excited by the magnetic stimulation, its ion channel state variables (*m, h, p*, and *n*) underwent a full cycle of Na channel activation/deactivation, P channel activation, and K channel activation (figure 9(C)), as observed in nodes that can sustain APs in a healthy, myelinated axon (figure 9(A)). Here, sufficient activation of the sodium channel (*m* variable) in node 56 was observed. However, the activation of the sodium channel (*m*) was more gradual (figure 9(C)) than that of a healthy axon (figure 9(A)) before the onset of the AP.

Figure 9(D) demonstrates the effects of microcoil stimulation on the individual channel current and state variables in the healthy, myelinated axon. Quick oscillations in the membrane potential led to rapid changes in the driving force. Therefore, the curves depicting the individual ion currents during the AP were unsmooth. Subthreshold coil stimulation did not significantly affect the state variables (*m, h, n*, and *p*) of various channels during an AP. We observed an abrupt and sufficient activation in the sodium channel (*m* = 1) and a slow decrease in the inactivating variable (*h*), similar to the case when the AP was initiated in node 55 in the absence of coil stimulation (figure 9(A)).

In conclusion, microcoil stimulation resulted in complete recovery of the channel dynamics in the targeted node, leading to the restoration of axonal conductance in the demyelinated axon.

#### 3.5.3. Restoration of axonal conductance by microcoil stimulation is not mediated by the diminished leak current in the demyelinated area

In the demyelinated axon, conductance failure occurred when the current leakage via the demyelinated area was substantial. Given that coil stimulation resumed axonal conductance, we questioned whether the leakage current via the demyelinated area (internode 55) somehow diminished during microcoil stimulation. To examine this possibility, we plotted the membrane current in node 55, internodes 55, and node 56, which are the three connected segments in the model.

**Figure 10.**
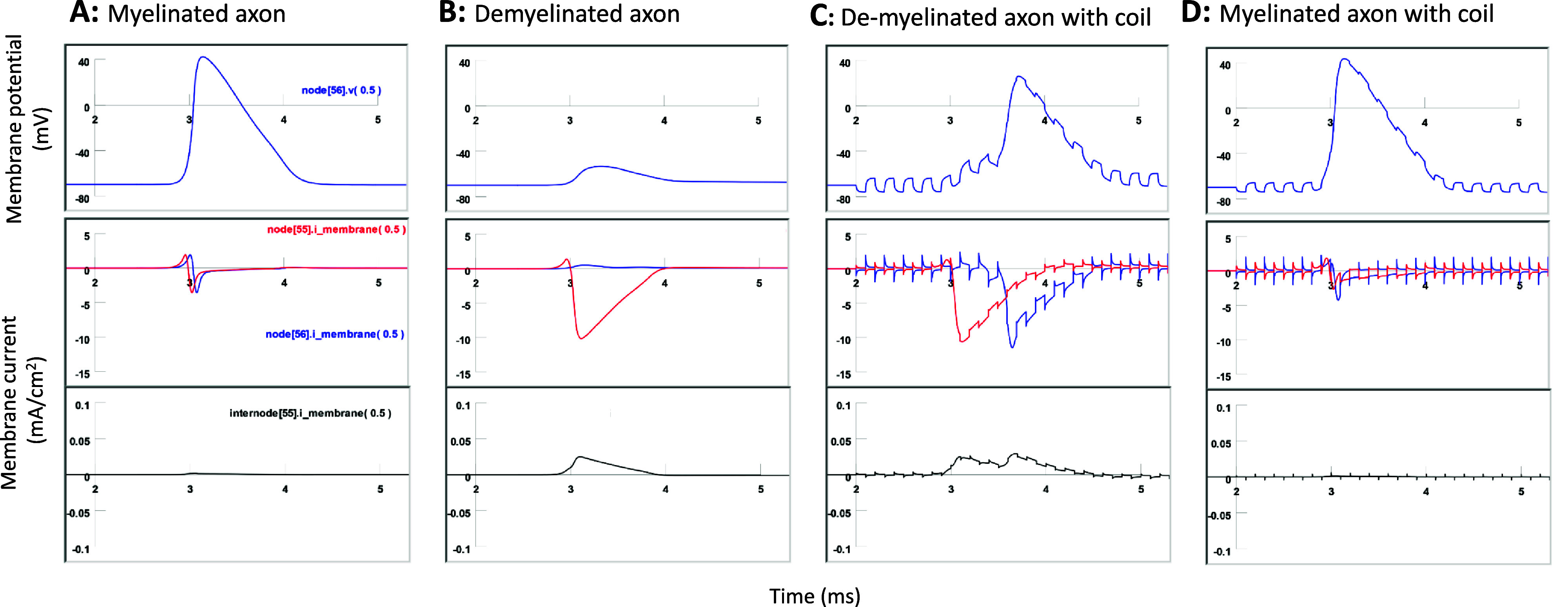
Microcoil stimulation does not prevent leakage in the demyelinated area. Plotted here are the membrane potential in node 56, and currents in the three connecting components (node 55, internodal 55, and node 56). The invading action potential is initiated by intracellular stimulation of node 0. (A). In a healthy, myelinated axon, leakage current (bottom trace) in internode 55 is zero. (B). Axon is demyelinated (Type II) between nodes 55 and 56. Large outward leakage in internode 55 is observed. The downstream node 56 receives minimal axial current from upstream node 55, leading to slight membrane depolarization. (C). When the demyelinated axon is under magnetic stimulation, the large outward leakage in internode 55 persists and even increases. The two peaks in the leakage current in internodal 55 are driven by the action potentials in node 55 and 56, respectively. (D). Healthy, myelinated axon under microcoil stimulation. There is no leakage current in the internodal 56 area.

When internode 55 was a myelinated segment in the healthy, myelinated axon, the membrane current leakage in internode 55 was zero (figure 10(A)). When demyelination occurred and the internode 55 became leaky, an outward leakage current was observed in internode 55, observed simultaneously with a large inward current in the upstream node 55 (figure 10(B)). This outward current in internode 55 originated from a portion of the inward current at node 55. The intensity of this leakage current appeared to be small (<0.02 mA cm^−2^). However, considering the large area provided by internode 55 (1000 *µ*m in length and 7 *µ*m in diameter), the leakage current could be substantial, leading to insufficient axial current to support depolarization and activation of the downstream node 56.

The coil stimulation led to the activation of node 56 with a significant delay (figure 10(C)). The APs in both nodes 55 and 56 were mediated by large inward currents in both respective nodes. Interestingly, each of these two large currents was pushed out of the leaky internode 55, forming a large, two-peak leaky current profile in internode 55 (figure 10(C)). Therefore, rather than observing a decreased leaky current from the demyelinated internode 55, we noticed an increased leakage current when axonal conductance was restored under microcoil stimulation. When the healthy axon was under coil stimulation, we observed zero leakage current from internode 55 (figure 10(D)).

In conclusion, the resumed axonal conductance by the microcoil stimulation in the demyelinated area is mediated by the depolarization of the targeted node. The magnetic stimulation on the targeted node prepared the node, so that the invading AP could easily activate them and the nerve pulses were able to be relayed. The mechanism described here is distinct from the well-known mechanism of remyelination-induced conductance restoration, which enhances node activation by reducing the current leakage in the demyelinated area.

## 4. Discussion

In this study, we tested the hypothesis that magnetic stimulation can restore the axonal conductance in locally demyelinated axons by activating the node in the demyelinated area. We built a multi-compartment NEURON model that contains the classic F–H channel dynamics and internodal segments, whose myelin thickness can be quantitatively controlled to simulate different levels of demyelination. This study builds on our understanding of the effects of demyelination on axonal function and the potential for targeted interventions using magnetic stimulation. The model-generated results are quantitatively in agreement with those reported in the literature. For example, our data demonstrated that significant loss of myelin thickness in a single internode can cause significant delay or complete failure of AP propagation (figure 4), which is consistent with that reported by (Koles and Rasminsky 1972). We also found that the presence of only a minimal extracellular myelin layer is sufficient in axonal conductance (figure 4). This is in agreeance with simulation results in (Hines and Shrager 1991) and experimental data from (Shrager and Rubinstein 1990). Using this demyelination model in combination with the microcoil model, this study produced several important findings.

First, activation of the node of Ranvier is a fundamental mechanism underlying saltatory conductance in myelinated axons. Therefore, methods that can enhance nodal excitability are thought to play an important role in preserving axonal conduction (Seidl 2014). Previously in a model work, Naud and Longtin (2019) found that the effects of demyelination on axonal conductance delay can be largely counterbalanced by increasing the excitability of nodes surrounding the demyelinated area. We demonstrated, *for the first time*, that microcoil stimulation could be used to enhance nodal activation and shorten the delayed nerve pulses traveling in the Type I demyelinated axons (figure 5). More importantly, we demonstrated that microcoil stimulation could restore function in Type II demyelinated axons, whose conductance was completely blocked. The rescued demyelinated axon could conduct the nerve pulses without noticeable delay, similar to a healthy, myelinated axon (figure 4(A)). The biophysics mechanism for the interaction between the node and the electromagnetic field is that the electromagnetic field provides a membrane potential oscillation whose frequency is defined by the stimuli. Although direct electrophysiology confirmation of node membrane potential under time-varying magnetic stimulation is lacking, we have demonstrated in our previous work, using intracellular recording from invertebrate neurons, that membrane potential oscillation is indeed orchestrated by microcoil stimulation (Ye and Barrett 2021, Ye *et al* 2024).

Second, we analyzed the impact of coil location, its stimulation amplitude, and frequency on the effectiveness of axonal restoration. Although results from these analyses are constrained by the parameters used in the models that describe the axon and the microcoil, respectively, they nevertheless generated several interesting insights for optimized stimulation practices. We report that restoring axonal conductance could be accomplished by subthreshold stimulation, suggesting that this neuromodulation method could yield minimal side effects (figures 5 and 6). We also found that direct targeting of the nodes in the demyelinated area was critical for the effective recovery of axonal function (figures 5 and 6). The future success of *µ*MS is dependent on the precise positioning and temporal control of the stimulus waveform of the microcoil in clinical practices, and these related technologies were developed quickly (Sekirnjak *et al* 2008, Bashir *et al* 2013). Finally, we studied the interaction between the high frequency stimulus and the physiological nerve signal in demyelinated axons, which provided a mechanistic interpretation of why stimuli of certain frequencies could be more successful than others in rescuing the demyelinated axons (figure 7). The parameter analysis is of significant value in the design and development of microcoil technology to treat demyelination disorders.

Third, a detailed analysis of the underlying currents in the nodes suggested that node activation, in combination with the axial current generated by the invading AP, allowed the AP to be relayed through the demyelinated area (figure 8). Notably, the presence of a weak axial current (which was generated by the invading AP) in the demyelinated axon (figure 8(C)) was essential for the restored AP with microcoil stimulation. Since the invading AP represents the timing of the physiological pulses, this result suggested that microcoil stimulation could provide a ‘virtual bridge’ to the demyelinated axon, and ensured a functional recovery of axonal conductance.

Fourth, we demonstrated that node activation and recovery of axonal conductance by magnetic stimulation were mediated by the resumed channel activation mechanisms, including sodium channel activation (figure 9). It is not mediated by any decrease in the leakage current in the internodal region (figure 10). Recent studies have indicated that specific sodium channel isoforms play an important role in MS during periods of remission, including restoration of impulse conduction after demyelination and axonal degeneration (Waxman 2006). An increase in the density of sodium ion channels has been linked to the improved propagation of demyelinated axons and membrane reconfiguration following demyelination (Ritchie *et al* 1981, Rasband *et al* 1998). Our results provide strong evidence that magnetic stimulation can directly target the sodium channels and boost their opening, leading to restoration of node activity. Future experiments using node patch clamp technology, which has recently been significantly improved (Kanda *et al* 2019), will test some of the predictions made from this modeling work.

### 4.1. Model limitations

By virtue of computational complexity, several assumptions were made to simplify the modeling process.

First, the myelinated axon was modeled as a simple geometry of a straight cylinder, without considering variabilities such as diameters, nerve undulation, and bending of the axon for local activation (Basser *et al* 1992, Abdeen and Stuchly 1994, Lu *et al* 2008). Our modification includes the electrical properties of the myelin but omits the F–H space between the myelin and the axolemma. This space provides a layer of resistivity outside the axolemma and may affect extracellular electric current flow if the axon was stimulated by an electrode. However, it should not affect the distribution of the induced electric field for axonal stimulation, since the magnetic field can penetrate the tissue without attenuation. More detailed axonal morphology is needed in future modeling endeavors.

Second, the demyelination process was applied uniformly to the entire internodal region. It has been recognized that a uniform reduction in myelin thickness offers only a very rough first approximation of the situation in real demyelinated fibers. Pathological changes may be found primarily in the paranodal regions rather than uniformly distributed throughout the internodes (Smith and Koles 1970), which causes axonal blockage or delay (Koles and Rasminsky 1972). To model paranodal demyelination, the internodal region will have to be divided into multiple segments and the loss of the axial current is exclusive to the paranodal segment of the internodal. Morphologically, this represents a moderate form of demyelination in comparison with our model, which simulates the complete internodal demyelination. We predict that microcoil activation of the node could be efficient in restoring the axonal conductance in paranodal demyelination.

Third, the F–H representation of node channels does not include several ion channels, such as Ca^2+^ and A-type K^+^ channels, which are essential for neural excitability (Tan *et al* 2013). The model, like many others, places the voltage-dependent ion channels only at the node of Ranvier, although low density Na channels are found in the internodal axolemma. Na channels may also diffuse from the node to the internode during demyelination (Eftekharpour *et al* 2007), though others suggest that Na channels are not free to diffuse from the node following demyelination (Shrager 1989). Na ion channel density on the internode is much lower than that on the node (Hines and Shrager 1991). Therefore, the internodal Na channel is unlikely be the target of microcoil stimulation, nor will it affect the passive current flow between the nodes. Therefore, this simplification will not challenge the major conclusion that axonal conductance restoration is due to the combined effects of coil-induced node depolarization and the incoming axial current.

K^+^ channels were modeled on the nodes, but not on the internodal, as in previous models (Mcintyre *et al* 2002) (Resnick *et al* 2018). However, it is known that K^+^ channels are present at the internode (Hines and Shrager 1991), and at a higher density than Na and P channels. A more recent model includes voltage-dependent K^+^ channels in juxtaparanode and internode regions (Sleutjes *et al* 2019). Future work shall investigate if the presence of internodal K^+^ channels will impact microcoil performance in rescuing axonal conductance.

Fourth, we only modeled focal demyelination in one internodal region of 1000 *µ*m, a dimension that allows the precise targeting of 1–2 nodes by one microcoil. If a longer region of demyelination is considered, one single microcoil could be insufficient to relay the AP that involved multiple nodes. We simulated the cases in which both sides of the myelin were removed for a single node, and found this exacerbated the axonal conductance. Complete demyelination happens when myelin thickness falls below 2% (in comparison, in single-side demyelination, complete blockage happens when myelin thickness decreases to 1.1%). Microcoil stimulation on this node is incapable of restoring conductance (data not shown). To activate multiple nodes to relay the nerve pulses, an array design of microcoils could provide a practical solution (Minusa *et al* 2019).

Fifth, intrinsic to the implementation of NEURON modeling of extracellular stimulation, the extracellular electric field was applied equally around each model compartment, without the consideration of the field distortion by the presence of axons. Furthermore, the extracellular voltage generated by the membrane current was neglected. Although such an approach is typical in the field (Joucla *et al* 2014, Ye and Steiger 2015), it could cause underestimation of the field generated by the miniature coil and introduce errors (Mcintyre *et al* 2004, Lee and Grill 2005).

Finally, future work will be needed to analyze the effects of axon length, axon diameter, and number of nodes of Ranvier on the restoration of axonal conductance by the microcoil stimulation. Various microcoil designs have emerged in the literature for neural stimulation (Ge *et al* 2024), and it is worthwhile to test various coil parameters (i.e. shape and size) and the capability of these coils in restoring axonal conductance using the platform we built here.

### 4.2. Potential clinical applications

This work suggests that microcoil stimulation could be used to promote functions in demyelinated axons. Due to the focality provided by the microcoil, the technology could be applied to local demyelination in central and peripheral nerve demyelination conditions.

Currently, several methods have been explored to generate electromagnetic fields for the treatment of demyelination problems. Deep brain stimulation (DBS) has been used to improve MS-related tremor (Brandmeir *et al* 2020). However, this is a surgical procedure that does not directly affect peripheral nerve demyelination. The surgery carries some risk, including bleeding in the brain and infection (Doshi 2011, Bjerknes *et al* 2014), lead migration, and electrode fracture (Jitkritsadakul *et al* 2017). Moreover, therapeutic effects can be largely altered by the inflammatory and immune responses due to the direct contact between the stimulating electrode and the tissue (Kim *et al* 2004, Liu *et al* 2017), and the formation of glial scarring around the individual electrode (Polikov *et al* 2005, Grill *et al* 2009), which leads to unpredictable changes or loss of neural response (Polikov *et al* 2005).

Repetitive TMS (rTMS) has shown potential as a treatment option for peripheral nerve demyelination, particularly in managing the pain associated with conditions like diabetic neuropathy (Xu and Xu 2021). TMS can promote remyelination of neurons by activating axonal fibers and increasing the number of oligodendrocytes (Cullen *et al* 2019, Nguyen *et al* 2024). This noninvasive method does not require the coil to be in direct contact with the target tissue (Maccabee *et al* 1991, 1993, Ye *et al* 2010, 2011, Ye and Steiger 2015). This mitigates numerous problems that can arise at the brain–electrode interface, such as charge transfer, electrode surface modification, and corrosion (Polikov *et al* 2005, Cogan 2008, Koivuniemi *et al* 2011). In rTMS, the induced electric current is defined by coil design and, therefore, could be better controlled. However, due to the large size of the coil and the fast decay of the induced electric field around the coil (Polk 1990, Polk and Song 1990), clinically used coils cannot provide DBS with high spatial resolution.

Transcranial direct-current stimulation (tDCS) applies electric currents from electrodes attached to the scalp. tDCS can directly stimulate peripheral nerves in the skin, potentially influencing their function and impacting demyelination processes occurring in those nerves. tDCS may have neuroprotective effects by modulating inflammatory responses (Callai *et al* 2022), which could be beneficial in conditions where demyelination is driven by inflammation. tDCS could also promote remyelination (Lee *et al* 2017, Rossi *et al* 2024). Nevertheless, in tDCS, the distribution of the electric field to the targeted neural tissue is affected by the inhomogeneity of the structure and could raise challenges for stimulation of focally demyelinated areas.

The last decade has witnessed the rapid development of *µ*MS technology for neural stimulation since the first report of the use of a commercial inductor for retinal ganglion neuron stimulation (Bonmassar *et al* 2012). Since then, the neuromodulatory effects of microcoils have been confirmed in various biological systems (Park *et al* 2013, Lee *et al* 2016, Lee and Fried 2017, Golestanirad *et al* 2018, Ye and Kaszuba 2019, Ye and Barrett 2021, Saha *et al* 2022a, Ye *et al* 2022a, 2024). One of the advantages of microcoil technology is that it can be implanted close to the target area for stimulation. Microcoil technology has been used for various nerve stimulations, including the vagus nerve (Jeong *et al* 2022, Saha *et al* 2024) and sciatic nerve (Saha *et al* 2023), for neural activation. This work is the first to reveal that *µ*MS could be used to target nodes for conductance recovery, and it provides detailed analysis of the coil parameters that are essential for the success of restoration. It is conceivable that *µ*MS can be used to stimulate these nerves under demyelinated conditions to maintain their functions.

Local demyelination occurs in many other situations. For example, axonal fibers are demyelinated, and nerve conductance is impaired in SCI (Ye *et al* 2012, Liu *et al* 2013). Peripheral nerve injury of the upper extremity commonly occurs in patients who participate in recreational and occupational activities (Neal and Fields 2010). Neuropraxia is the mildest form of traumatic peripheral nerve injury. It is characterized by focal segmental demyelination at the site of injury without disruption of axon continuity and its surrounding connective tissues. This condition results in blockage of nerve conduction and transient weakness or paresthesia. Complete recovery is expected upon spontaneous remyelination in several months when the nerve completes remyelination. Our data demonstrates that *µ*MS could be effective if a fine-tuned stimulation protocol is available to accommodate varying degrees of demyelination/remyelination, before the remyelination is completed.

Another application of *µ*MS technology is to develop the microcoil as CIs to help patients with severe hearing loss by electrically stimulating the auditory nerve. Lee *et al* (2022) have used *µ*MS to generate efficient and spatially precise activation of the auditory system, possibly due to the fact that the microcoil can confine the electric field more narrowly than conventional microelectrodes. This new method opens interesting possibilities to improve the resolution of auditory restoration by implanted devices. However, following sensorineural deafening, acute and progressive SGN undergo ultrastructural changes, including axon demyelination (Tagoe *et al* 2014), causing delay and blockage of axonal conductance (Resnick *et al* 2018). Results from this paper suggest that it is possible to compensate for these pathological changes by adjusting the stimulation protocol for node activation.

### 4.3. Future efforts in developing electromagnetic stimulation as an alternative strategy for demyelination problems

This work provides a novel strategy to restore axonal conductance in demyelinated axons with electromagnetic stimulation. Several unanswered scientific questions should be addressed before this method could be developed for clinical application.

First, the functions of the nervous system depend on the precise timing of the nerve pulses. Precise regulation of nerve conduction velocity is required for correct exertion of motor skills, sensory integration, and cognitive functions. Although we successfully restored axonal conductance in the demyelinated axon using magnetic stimulation, further research is needed to explore the possibility of precise control over the timing of neural activity. For example, a delay and distortion of the AP still existed when it traveled across the demyelinated area, even under strong microcoil stimulation in Type I demyelinated axons (figure 5). It would be beneficial to investigate what the parameters in the magnetic stimulus can best counterbalance the deficit in AP propagation and produce minimized delay and jittering.

Second, demyelination/remyelination is a dynamic process in many pathological conditions. This paper demonstrates that the rescue effects of coil stimulation can vary depending on the degree of demyelination. The effectiveness of restoration in severely demyelinated axons is likely to depend on an optimal combination of the amplitude, frequency, and orientation of the coil and arrangement of coils (coil array). Therefore, to fully take the advantage of the microcoil stimulation in dealing with complicated demyelination conditions, further research must take this dynamic change into consideration in the technique design.

Third, many examples have suggested that, using a novel design of electrode arrays, it is likely to generate targeted, focal stimulation of the neural tissue (Gaunt *et al* 2009, Huan *et al* 2021). Electrical stimulation delivered with various types of electrodes or electrode arrays may also assist the conductance after demyelination. Further research shall improve the specificity and focality of *µ*MS or a microarray of electrodes to fully harvest their capabilities in treating local demyelination conditions.

## 5. Conclusion

This work presents the first evidence that the electromagnetic field generated by microcoil stimulation can restore conductance in the demyelinated axons. We also reveal the novel biophysics mechanisms underlying the interaction between the microcoil and the nodes of Ranvier for such functional recovery. Neural activation with electromagnetic field could be a promising alternative in the treatment of demyelination conditions.

## Data Availability

All data that support the findings of this study are included within the article (and any supplementary files).

## Appendix A F–H model

The membrane potential on the node of Ranvier (*V*_m_) is given by

\begin{align*}\frac{{{\text{d}}{V_{\text{m}}}}}{{{\text{d}}t}} = \frac{{{I_{\text{c}}}}}{{{C_{\text{m}}}}}\end{align*}

where *I*_c_ is the capacity current and *C*_m_ is the membrane capacitance. *I*_c_ is computed as the difference between the total membrane current (*I*_membrane_) and the four ion channel currents. *I*_membrane_ is caused by the axial current flowing into the node compartment,

\begin{align*}{I_{\text{c}}} + {I_{{\text{Na}}}} + {I_{\text{K}}} + {I_{\text{p}}} + {I_{\text{L}}} = {I_{{\text{membrane}}}}\end{align*}

where *I*_Na_ is the sodium current, *I*_K_ is the potassium current, *I*_p_ is the non-specific current, and *I*_L_ is the leakage current (*µ*A cm^−2^),

\begin{align*}{I_{{\text{Na}}}} &amp; = {P_{{\text{Na}}}}h{m^2}\frac{{E{F^2}}}{{RT}}\frac{{[ {{\text{Na}}{]_{\text{o}}} - } [{\text{Na}}{]_{\text{i}}}{\text{exp}}\left( {\frac{{EF}}{{RT}}} \right)}}{{1 - {\text{exp}}\left( {\frac{{EF}}{{RT}}} \right)}}\end{align*}

\begin{align*}{I_{\text{K}}} &amp; = {P_{\text{K}}}{n^2}\frac{{E{F^2}}}{{RT}}\frac{{[ {{\text{K}}{]_{\text{o}}} - } [{\text{K}}{]_{\text{i}}}{\text{exp}}\left( {\frac{{EF}}{{RT}}} \right)}}{{1 - {\text{exp}}\left( {\frac{{EF}}{{RT}}} \right)}}\end{align*}

\begin{align*}{I_{\text{P}}} &amp; = {P_{\text{P}}}{p^2}\frac{{E{F^2}}}{{RT}}\frac{{[ {{\text{Na}}{]_{\text{o}}} - } [{\text{Na}}{]_{\text{i}}}{\text{exp}}\left( {\frac{{EF}}{{RT}}} \right)}}{{1 - {\text{exp}}\left( {\frac{{EF}}{{RT}}} \right)}}\end{align*}

\begin{align*}{I_{\text{L}}} &amp; = {g_{\text{L}}}\left( {V - {E_{\text{L}}}} \right).\end{align*}

Here, ${ }E = {V_{\text{m}}} + {E_{\text{r}}}$. *E* is the absolute membrane potential, and *E*_r_ is the membrane resting potential. [Na]_i_ and [Na]_o_ are sodium concentrations inside and outside the axon, respectively. *E*_L_ denotes the reversal potential of leakage channel. [K]_i_ and [K]_o_ are the potassium concentrations inside and outside the axon, respectively. *F* is Faraday’s constant, *R* is the gas constant, and *T* is the absolute temperature in Kelvin. *P*_Na_ and *P*_K_ are the sodium and potassium permeability constants, respectively. *P*_p_ is the non-specific permeability constant and *g*_L_ is the leak conductance.

The variables *m, h, n*, and *p* are the gating variables of various ion channels. Specifically, *m* and *h* are the activation and inactivation variables for the sodium channel, respectively; *n* and *p* are the activation variables for the potassium and nonspecific channels. They are computed using ordinary differential equations (Frankenhaeuser and Huxley 1964),

\begin{align*}\frac{{{\text{d}}m}}{{{\text{d}}t}} &amp; = {\alpha _{\text{m}}}\left( {1 - m} \right) - {\beta _{\text{m}}}m\end{align*}

\begin{align*}\frac{{{\text{d}}h}}{{{\text{d}}t}} &amp; = {\alpha _{\text{h}}}\left( {1 - h} \right) - {\beta _{\text{h}}}h\end{align*}

\begin{align*}\frac{{{\text{d}}n}}{{{\text{d}}t}} &amp; = {\alpha _{\text{n}}}\left( {1 - n} \right) - {\beta _{\text{n}}}n\end{align*}

\begin{align*}\frac{{{\text{d}}p}}{{{\text{d}}t}} &amp; = {\alpha _{\text{p}}}\left( {1 - p} \right) - {\beta _{\text{p}}}p\end{align*}

where the *α* and *β* terms are functions of membrane potential (*V*_m_).

## Appendix B Calculation of the induced electric field for neural stimulation

A magnetic field is generated around the coil when a voltage pulse (*V*) is delivered to the coil. The voltage across the coil is equal to the voltage drop due to the coil resistance and inductive impedance,

\begin{align*}V = IR + L\frac{{{\text{d}}I}}{{{\text{d}}t}}\end{align*}

where $I$ is the current in the coil, *R* is the coil resistance, and *L* is coil inductance.

For the rising phase of the pulse, solution of the coil current is

\begin{align*}I = \frac{V}{R}\left( {1 - {{\text{e}}^{ - \frac{{tR}}{L}}}} \right).\end{align*}

Therefore, $I{\text{ }}$ is zero at the beginning of the pulse and increased exponentially to a plateau value (*V*/*R*). For the falling phase of the pulse, the coil current is

\begin{align*}I = \frac{V}{R}{{\text{e}}^{ - \frac{{tR}}{L}}}.\end{align*}

Therefore, the coil current decays exponentially in the falling phase, from the maximum (*V*/*R*) to zero.

In the coordination system illustrated in figure 2(A), with some mathematical calculation, the electric field induced by the time-varying coil current (figure 2(B)) has been calculated elsewhere (Ye 2022, Ye *et al* 2022a).

For the onset of the pulse,

\begin{align*}{E_x} = - \frac{{V{\mu _0}N{R_{\text{c}}}{{ }^2}}}{{2Ll}}\frac{y}{{{x^2} + {y^2}}}{{\text{e}}^{ - \frac{{tR}}{L}}}\end{align*}

\begin{align*}{E_y} = \frac{{V{\mu _0}N{R_{\text{c}}}{{ }^2}}}{{2Ll}}\frac{x}{{{x^2} + {y^2}}}{{\text{e}}^{ - \frac{{tR}}{L}}}.\end{align*}

For the offset of the pulse,

\begin{align*}{E_x} = \frac{{V{\mu _0}N{R_{\text{c}}}{{ }^2}}}{{2Ll}}\frac{y}{{{x^2} + {y^2}}}{{\text{e}}^{ - \frac{{tR}}{L}}}\end{align*}

\begin{align*}{E_y} = - \frac{{V{\mu _0}N{R_{\text{c}}}{{ }^2}}}{{2Ll}}\frac{x}{{{x^2} + {y^2}}}{{\text{e}}^{ - \frac{{tR}}{L}}}\end{align*}

where *E_x_* and *E_y_* are the *x* and *y* components of the magnetically induced electric field, respectively. In addition, ${R_{\text{c}}}$ is the radius of the coil, *L* is the inductance of the coil, *R* is the resistance of the coil, *N* is the number of loops, and *l* is the coil length. *Μ*_0_ = 4*π* × 10^−7^ H m^−1^ is the vacuum permeability.

For the onset of the pulse, the electric potential distribution along the axon is expressed as

\begin{align*}V\left( x \right) = {\mathop \int \nolimits }{E_x}\left( x \right){\text{d}}x = - \frac{{V{\mu _0}N{R_{\text{c}}}{{ }^2}}}{{2Ll}}{\text{atan}}\left( {\frac{x}{y}} \right){{\text{e}}^{ - \frac{{tR}}{L}}}.\end{align*}

The electric potential distribution along the axon for the pulse offset is expressed as

\begin{align*}{\text{V}}\left( {\text{x}} \right) = {\mathop \int \nolimits }{{\text{E}}_{\text{x}}}\left( {\text{x}} \right){\text{dx}} = \frac{{{\text{V}}{{{\mu }}_0}{\text{N}}{{\text{R}}_{\text{c}}}{{\text{ }}^2}}}{{2{\text{Ll}}}}{\text{atan}}\left( {\frac{{\text{x}}}{{\text{y}}}} \right){{\text{e}}^{ - \frac{{{\text{tR}}}}{{\text{L}}}}}.\end{align*}

We calculated the gradient of the electric field along the axon, since gradient of the extracellular electric field is used as an indicator of membrane depolarization/hyperpolarization (Ranck 1975, Rattay 1986, 1999). Since the microcoil generates a local electric field, the local gradient could be large enough even if the electric field is less significant. As such, computation of the gradient of the electric field has been used to design and position the coil for neural stimulation (Lee *et al* 2016, 2019).

For the onset of the pulse, the field gradient along the axon was

\begin{align*}\frac{{\partial {E_x}}}{{\partial x}} = - \frac{{V{\mu _0}N{R_{\text{c}}}{{ }^2}}}{{Ll}}xy{\left( {{x^2} + {y^2}} \right)^{ - 2}}{{\text{e}}^{ - \frac{{tR}}{L}}}.\end{align*}

For the offset of the pulse, the field gradient along the axon was

\begin{align*}\frac{{\partial {E_x}}}{{\partial x}} = \frac{{V{\mu _0}N{R_{\text{c}}}{{ }^2}}}{{Ll}}xy{\left( {{x^2} + {y^2}} \right)^{ - 2}}{{\text{e}}^{ - \frac{{tR}}{L}}}.\end{align*}

The ‘neutral point’ was a point on the axon where the field gradient is zero. From (B10) and (B11), this point was found to locate at *x* = 0. If the coil current was in the clockwise direction and was decreasing over time (offset phase), the induced electric field was clockwise in direction (figure 2(C)). Points for peak depolarization and hyperpolarization were solved by ${ }\frac{{\text{d}}}{{{\text{d}}x}}\left( {\frac{{\partial {E_x}}}{{\partial x}}} \right) = 0$. The point for peak depolarization was at

\begin{align*}x = y/\sqrt 3 .\end{align*}

The location of peak hyperpolarization was at

\begin{align*}x = - y/\sqrt 3 .\end{align*}

Note these locations were defined by the coil’s distance to the axon, suggesting that the location of maximal polarization is defined by the distance between the coil and the axon. This information will be comprehensively used to position the coil for targeting the nodes.
